# Cheminformatics analysis of chemicals that increase estrogen and progesterone synthesis for a breast cancer hazard assessment

**DOI:** 10.1038/s41598-022-24889-w

**Published:** 2022-11-30

**Authors:** Alexandre Borrel, Ruthann A. Rudel

**Affiliations:** Silent Spring Institute, Newton, MA USA

**Keywords:** Computational biology and bioinformatics, Risk factors, High-throughput screening, Breast cancer

## Abstract

Factors that increase estrogen or progesterone (P4) action are well-established as increasing breast cancer risk, and many first-line treatments to prevent breast cancer recurrence work by blocking estrogen synthesis or action. In previous work, using data from an in vitro steroidogenesis assay developed for the U.S. Environmental Protection Agency (EPA) ToxCast program, we identified 182 chemicals that increased estradiol (E2up) and 185 that increased progesterone (P4up) in human H295R adrenocortical carcinoma cells, an OECD validated assay for steroidogenesis. Chemicals known to induce mammary effects in vivo were very likely to increase E2 or P4 synthesis, further supporting the importance of these pathways for breast cancer. To identify additional chemical exposures that may increase breast cancer risk through E2 or P4 steroidogenesis, we developed a cheminformatics approach to identify structural features associated with these activities and to predict other E2 or P4 steroidogens from their chemical structures. First, we used molecular descriptors and physicochemical properties to cluster the 2,012 chemicals screened in the steroidogenesis assay using a self-organizing map (SOM). Structural features such as triazine, phenol, or more broadly benzene ramified with halide, amine or alcohol, are enriched for E2 or P4up chemicals. Among E2up chemicals, phenol and benzenone are found as significant substructures, along with nitrogen-containing biphenyls. For P4up chemicals, phenol and complex aromatic systems ramified with oxygen-based groups such as flavone or phenolphthalein are significant substructures. Chemicals that are active for both E2up and P4up are enriched with substructures such as dihydroxy phosphanedithione or are small chemicals that contain one benzene ramified with chlorine, alcohol, methyl or primary amine. These results are confirmed with a chemotype ToxPrint analysis. Then, we used machine learning and artificial intelligence algorithms to develop and validate predictive classification QSAR models for E2up and P4up chemicals. These models gave reasonable external prediction performances (balanced accuracy ~ 0.8 and Matthews Coefficient Correlation ~ 0.5) on an external validation. The QSAR models were enriched by adding a confidence score that considers the chemical applicability domain and a ToxPrint assessment of the chemical. This profiling and these models may be useful to direct future testing and risk assessments for chemicals related to breast cancer and other hormonally-mediated outcomes.

## Introduction

People are exposed every day to numerous chemicals that can affect the body’s hormone systems. These endocrine disrupting chemicals (EDCs) are found in diet (food and water), consumer products, drugs, industrial pollutants, and pesticides. Daily exposure to a cocktail of these chemicals raises concerns for increased risk of various outcomes, including hormone-dependent cancers^1^.

Breast cancer is a worldwide concern and the most common invasive malignancy in the US^2^. The majority of breast cancers are hormone-responsive and classified as estrogen receptor positive^3^. The relationship between hormone exposure and breast cancer is well documented in experimental animals and humans. In both rats and mice, hormone supplementation increases mammary tumors, and in humans, ovariectomy reduces risk while hormone replacement therapy increases it^4^. First line treatments to reduce the risk of breast cancer recurrence in postmenopausal women use aromatase inhibitors to reduce tissue estradiol (E2) levels^5^. Furthermore, the concentration of E2 in breast tissues of postmenopausal woman is higher in cancerous tissues than in benign tissues by a median factor of 2.5-fold^6^, suggesting that localized production of E2 is an important factor in breast cancer risk.

Until recently, research on EDCs in relation to breast cancer risk has focused on chemicals that bind and activate the estrogen receptor (ER), mimicking estrogen. However, another potential pathway could be from chemicals that do not directly interact with ER but instead modulate steroidogenesis pathways to increase the level of E2 and P4 could also promote breast cancer development and progression^5,7–10^.

A previous study from our institute used publicly available data and identified 296 chemicals that increased E2 (182), P4 (185) or both (71) from 2,012 chemicals tested in the HT-H295R assay as part of US EPA’s Tox21/ToxCast program^5^. This assay is internationally validated for regulatory contexts by the Organization for Economic Co-operation and Development (OECD)^11,12^ and slight variations are also used for research purposes^13–18^. We have shown that chemicals known to cause mammary gland effects in vivo are enriched for increasing E2 and P4 steroidogenesis^5^, so exposure to these chemicals may also increase breast cancer risk. Specifically, among the 2,012 chemicals tested in the assay, 45 are known to cause mammary gland effects in vivo, and 64% of these (29 chemicals) increased E2 or P4 synthesis, including the well-known mammary carcinogen 7,12-dimethylbenz(a)anthracene^5^. In addition, there are 267 chemicals that cause mammary gland tumors in experimental studies in vivo, but that haven’t been evaluated to see whether they affect steroidogenesis.

Given the many thousands of chemicals in commerce, in silico/computational approaches to identify chemicals that might increase E2 or P4 would support additional testing and exposure reduction which could prevent breast cancer. We are not aware of any structural model/computational model that has been developed to identify molecules likely to increase E2 or P4. Quantitative structure–activity relationship (QSAR) models developed using artificial intelligence (AI) and machine learning approaches offer the opportunity to rapidly screen a large set of chemicals to help identify those that may increase E2 or P4 and subsequently breast cancer risk.

In this paper, we used the HT-H295R screening data for 2,012 chemicals, along with the previously published list of the chemicals that increased E2 and P4 steroidogenesis in this assay^5^, to develop cheminformatics tools that can identify chemicals likely to increase production of E2 and P4. First, we used a structure-based clustering on molecular descriptors and toxicity based chemotypes (ToxPrint). In the second step, we developed a QSAR model to predict the potential for a chemical to increase E2 or P4 based on structure. Finally, in order to identify modes of action, we applied the models to a set of 194 chemicals that cause mammary gland tumors but were not tested in H295R and predicted whether they are likely to increase E2 or P4 steroidogenesis.

## Material and methods

This study does not report experiment on human, does not involve human samples and does not report experiment on vertebrates.

### HT-H295R steroidogenesis assay

The HT-H295R steroidogenesis assay was used as a part of the ToxCast program developed by the US EPA^19^. The results of this assay were first reported in Karmaus et al. and Haggard et al.^20–22^. In these experiments, 2012 chemicals were tested on human H295R adrenocortical carcinoma cells to evaluate effects on steroidogenesis. Chemicals were selected from the ToxCast program phase I, II, and III, which include a wide range of chemicals including many potential EDCs and chemicals with a regulatory interest. Tested chemicals include many from exposure sources such as pesticides, diet, industrial, consumer and pharmaceutical products.

Because we are interested in breast cancer, we previously analyzed HT-H295R data to identify (i) chemicals that increase the level of estradiol (E2up) and (ii) chemicals that increase the level of progesterone (P4up)^5^. Briefly, H295R cells were treated with test chemicals at a single non-cytotoxic dose and concentrations of thirteen steroid hormones were measured in the culture media using high performance liquid chromatography with tandem mass spectroscopy (HPLC–MS/MS), and hit-calls were derived from ToxCast pipeline package in R (tcpl; http://epa.gov/ncct/toxcast/data.html)^20^. After this initial screen of 2012 chemicals, those that significantly affected four or more hormones (N = 656) were then tested again using a six-point concentration–response (CR) format^21^. As described Cardona and Rudel^5^, authors took several measures to optimize the balance between Type I (false positive) and Type 2 (false negative) errors. The approaches used rely heavily on EPA’s analysis of these same H295R data^21^, and are consistent with OECD and EPA test guidelines as well^11^. E2 and P4-up chemicals were first selected based on significantly increasing these hormones by OECD criteria (two consecutive concentrations and/or maximum non-cytotoxic concentration significantly different from control by ANOVA), as reported in Haggard 2018. Then, to minimize false positives, our list of active E2-up (n = 182) and P4-up (n = 185) chemicals had to meet three additional criteria—they had to produce a hormone response with a fold-change ≥ 1.5, and had to significantly perturb multiple components of the steroidogenesis pathway based on the calculated adjusted maximal mean Mahalanobis distance (adj.maxmMd > 0). The calculations for the adj.maxmMd were conducted by US EPA and are published in Haggard et al.^21^. We applied a third criterion for potency so that we only considered the most potent chemicals (lowest effective concentration ≤ 33 μM) as active. In addition to finding 182 E2-up and 185 P4-up chemicals that meet these criteria, authors also identified another set of chemicals, including 84 E2-up and 90 P4-up, as borderline-actives because they significantly increased E2 and P4 using the ANOVA-based method but did not meet the additional three criteria above. These are more likely to include some false positives, but there may be some true positives among them as well.

For the purposes of QSAR modeling, we excluded borderline-active chemicals and those that only increased E2 or P4 in the single dose assay from both the active and the inactive categories, since it is unclear whether they are active or not.

### Mammary carcinogen chemical set (MC)

We applied our QSAR models on a combined set of 267 rodent mammary carcinogens (MCs). This set has been compiled in our institute as an update of previous work^23^ (publication submitted). Before we applied our predicting models, we removed chemicals that were included in the training set.

### Chemical preparation and dataset curation

As the first step, chemical structures for 2012 chemicals tested in H295R and for 267 rodent mammary carcinogens were downloaded in SMILES string format from the EPA chemicals dashboard^24^ using their Chemical Abstract Services Registry Number (CASRN). Structure preparation and curation followed the best practices in the field^25,26^. It was performed by using following steps: removal of hydrogen atoms, removal of any metal ions, checking stereochemistry, desolvation, and removal of salt fragments. Finally, 87 chemicals from the initial set of 2012, including mixtures, ions and chemicals without known structure listed in the EPA chemical dashboard were excluded because they did not have a clear structures. Curation was realized using the RDKit (v.2021) python library integrated in our personal library CompDesc (v.0.21) available on TestPyPi platform (https://test.pypi.org/project/CompDesc/).

From each curated structure, a set of 616 1D and 2D descriptors was computed using the same packages developed in Python 3.9. The full list of descriptors is available on the CompDesc GitHub repository (https://github.com/ABorrel/CompDESC). An additional set of 19 physicochemical descriptors were predicted on each chemical using OPERA models (v.2.7)^27^.

Only informative and non-correlated descriptors were selected from the initial set of descriptors. First, descriptors having a null variance or the same value for more than 90% of the chemicals were removed. Subsequently, for the remaining descriptors, pairwise Pearson’s correlation coefficients were computed. Descriptors were clustered based on pair-wise correlation > 0.9 and only one descriptor from each cluster was randomly selected for further analysis. Descriptor selection and curation were realized in R (4.1.2).

### Self-organizing map (SOM)

The initial set of curated chemicals (N = 1925) was clustered into 64 clusters using the selected descriptors and the self-organizing map (SOM) algorithm^28^. The number of clusters was empirically chosen to achieve a balance between cluster number and the number of chemicals by cluster (see Supporting Information Fig. S1).

Clustering was developed using the R (v.4.1.2) with libraries *kohonen (v.3.0.10), factoextra (v.1.0.7), ggtree (v.3.0.2), ape (v.5.5) and phangorm (v.2.7.1)*.

### Chemotype: ToxPrint

A chemotype representation of each chemical was computed using ToxPrint blocks^29^ with a batch search on the EPA chemical dashboard^24^. Each chemical can be represented as a combination of chemotypes (i.e. chemical features called ToxPrint). Seven hundred and twenty-nine ToxPrints have been developed specifically for environmental and toxic chemicals using a set of structural features targeted to cover chemical structures from the large toxicity databases and regulatory inventories. They can be chemical substructures, bond types or specific elements.

### Structure similarity

Similarity between pairs of chemical structures was computed using Molecular ACCess System (MACCS) substructure 2D fingerprints and a Tanimoto score between 0 and 1, with a score of 1 indicating the chemicals are identical. Structure similarity functions are available in the RDKit (v.2021) python package (https://www.rdkit.org/).

### QSAR modeling

Classification QSAR models were developed to discriminate E2up-P4up chemicals vs. chemicals that do not impact these hormone levels. The QSAR modeling workflow was conducted according to the best practices in the fields^30–32^.

#### Machine learning

Five machine learning-based approaches were used to generate QSAR classification models to predict the potential for chemicals to be E2up or P4up: (i) classification and Regression Tree (CART)^33^; (ii) neural network (NN)^34^; (iii) Support Vector Machine (SVM) with a linear, radial and sigmoid kernel^35^; (iv) Random Forests (RF)/balanced RF^36^; and (v) Linear Discriminant Analysis (LDA) based on Fisher’s linear discriminant method. These five approaches were chosen to cover a large set of methods including linear and non-linear approaches. QSAR models were built using R (v.4.1.2) with packages *pls (v.2.7.3), randomForest (v.4.6.14), rpart (v.4.1.15), e1071 (v.1.7.8), nnet (7.3.17)* and *caret (6.0.88)*. In addition to the classic machine learning approaches, we developed a deep neural network (DNN) classification model^37^. The models were built using Keras (v.2.3.1) python deep learning library with TensorFlow 2.1.0 (2020) in Python (v.3.9). Different RF models were developed in Python 3.9 using the package *sklearn (v0.24.2)*. Hyperparameters were optimized using a grid optimization on five-fold cross-validation.

A similar protocol to that applied in Borrel et al.^38^ was used. Briefly, each model was tuned via grid optimization as appropriate for the machine learning algorithm, and parameters/models were chosen to maximize ten-fold cross-validation performance using the Matthew’s correlation coefficient (MCC). MCC criteria is considered optimal to analyze QSAR model performance as it represents the correlation between the observed and predicted classification with value ranges from − 1 (random prediction) to + 1 (perfect prediction), and it is a statistical metric that is least affected by imbalance in the dataset. Supplementary Information Table S1 reports grids of optimization and parameters and hyperparameters chosen.

#### Unbalanced set

Considering the imbalance between the number of active (< 10%) and inactive chemicals (> 90%) for RF, RF-balanced and DNN the Generalized tHreshOld ShifTing Procedure (GHOST) method was used to optimize the probability cutoff between predicted active and inactive^39^. QSAR models were also trained using an under-sampling approach with similar performance as the GHOST approach. Results for under-sampling are not reported here.

#### Evaluation of the classification model performance

The generated models were evaluated for their performance by calculating the number of true positives (TP), true negatives (TN), false positives (FP) and false negatives (FN). TP are the EDCs that increase E2 or P4 and are correctly predicted, TN are the inactives that are predicted as inactive by a model, FP represents inactive chemicals predicted as E2up or P4up, and FN represents the E2up and P4up chemicals incorrectly predicted as inactive. From those numbers, performance was computed using sensitivity (SE), specificity (SP), overall prediction accuracy (Acc), balanced accuracy (bAcc, average of sensitivity and specificity) and the MCC:

$$SE= \frac{TP}{TP+FN}$$

$$SP= \frac{TN}{TN+FP}$$

$$Acc= \frac{TP+TN}{TP+TN+FP+FN}$$

$$b\mathrm{Acc}= \frac{SE+SP}{2}$$

$$MCC= \frac{TP.TN-FP.FN}{\sqrt{(TP+FP)(TP+FN)(TN+FP)(TN+FN)}}$$

### Literature search

To confirm predicted E2up and P4up chemicals from the MC list, we conducted a literature search for each chemical predicted to be active. The search was performed on PubMed using the Python 3.9 library *Biopython (v.1.79)*. For each chemical, a generic search request was developed using MESH terms with chemical name and CAS ID and with general terms relevant to steroidogenesis. For E2up the generic search request was: “((((((("<*CASRN of chemical*>" OR "<*chemical name*>")) AND ("estradiol")) OR ((("<*CASRN of chemical*>" OR "<*chemical name*>")) AND ("steroidogenesis"))) OR ((("<*CASRN of chemical*>" OR "<*chemical name*>")) AND ("steroidogenic")))” and for P4up “((((((("<*CASRN of chemical*>" OR "<*chemical name*>")) AND ("progesterone")) OR ((("<*CASRN of chemical*>" OR "<*chemical name*>")) AND ("steroidogenesis"))) OR ((("<*CASRN of chemical*>" OR "<*chemical name*>")) AND ("steroidogenic")))”.

For each chemical, the number of articles was extracted with the article title. An initial manual screen was performed by subject matter experts in endocrine toxicology to select relevant publications based on likelihood that the study reports an outcome of altered serum or tissue E2 or P4 or a potentially related downstream effect, and abstracts were extracted for a further investigation.

All of the scripts, initial datasets and descriptors tables for this project are available on GitHub: https://github.com/SilentSpringInstitute/StructuralAnalysisE2upP4up.

## Results

### Composition of the dataset

**Figure 1:**
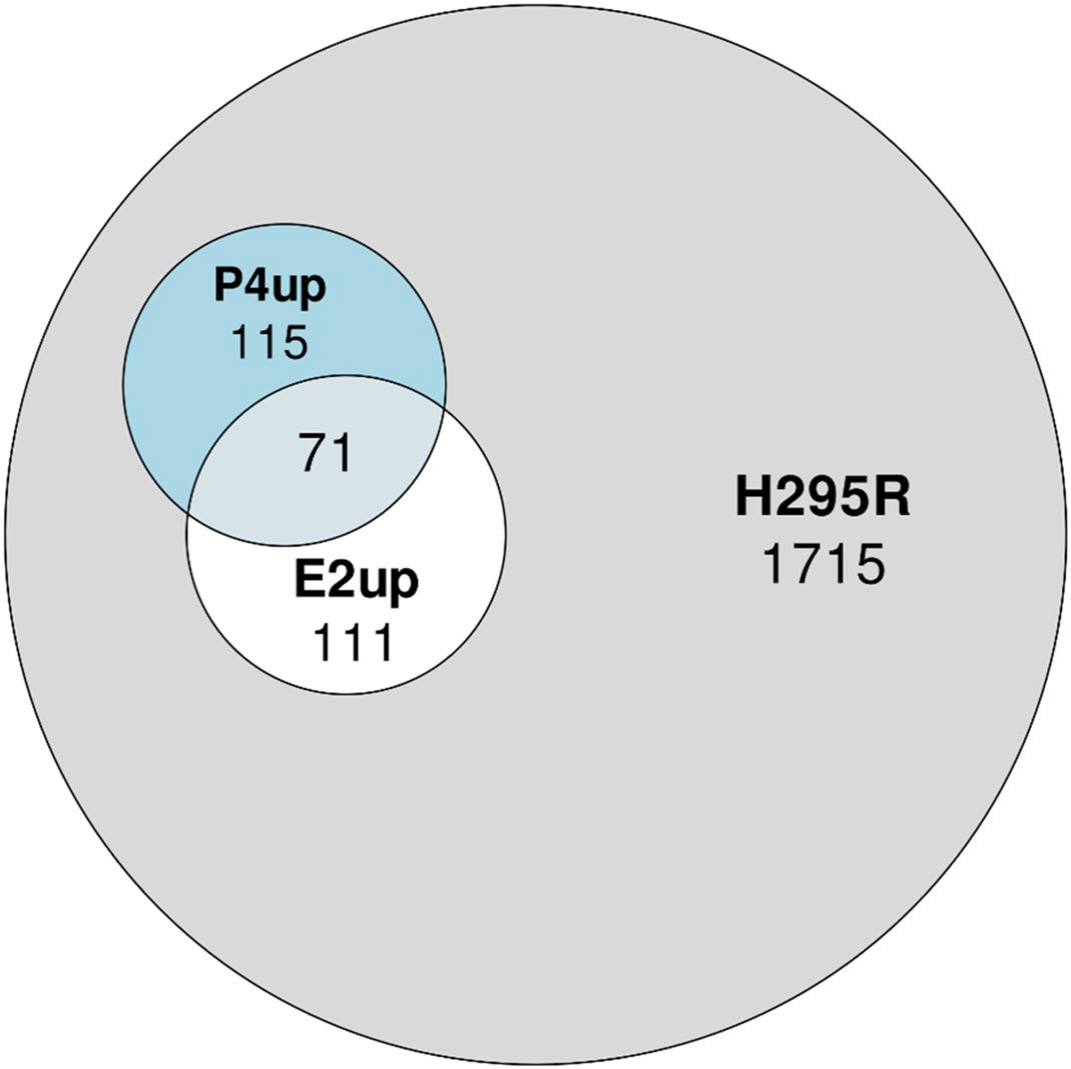
Venn diagram representing the overlap between the active chemical set that increases production of E2 (E2up), the active chemical set that increase production of P4 (P4up) and all chemicals tested in the H295R assay (H295R set).

The initial set of chemicals tested in H295R (H295R set) included 2012 chemicals. Of these, as described in Cardona and Rudel^5^, 182 increased E2 (E2up) and 186 increased P4 (P4up) in CR, with 71 increasing both (Fig. 1).

**Figure 2:**
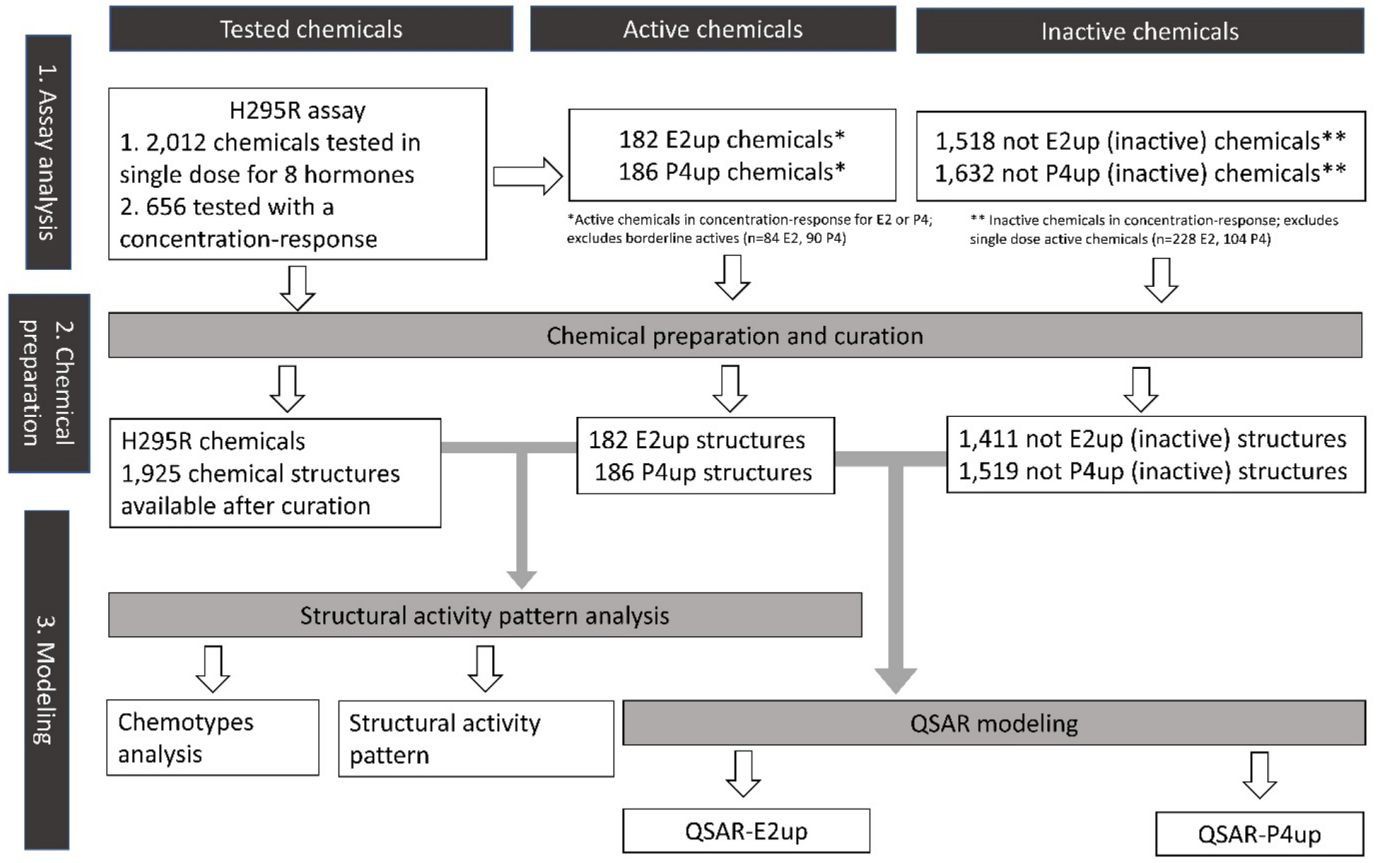
Workflow of the study.

To prepare the dataset for QSAR modeling, we removed 312 and 184 chemicals that were found active in the single dose testing, for E2 and P4 respectively, but inactive for the curve response assay testing. This step ensures that the chemical set defined as inactive did not include these possible or weak E2 and P4up chemicals (see “Material and methods” for detail). The Fig. 2 shows the workflow of this project. Table 1 shows the number of chemicals removed and the size of the final dataset used for QSAR modeling, before structure preparation.

**Table 1:** Summary of chemical sets used for this work.

**Chemical sets for the structural analysis (SOM and ToxPrint analysis)**	
Full set of chemicals (H295R)	2012
Active chemicals in concentration response curve for E2 (E2up)^a^	182
Active chemicals in concentration response curve for P4 (P4up)^a^	186
Borderline active chemicals excluded from the E2up active set^a^	84
Borderline active chemicals excluded from the P4up active set^a^	90
**Chemical sets used for the QSAR modeling**	
**Active**	
Active chemicals in concentration response curve for E2 (E2up)^a^	182
Active chemicals in concentration response curve for P4 (P4up)^a^	186
**Excluded due to uncertain activity**	
Borderline active chemicals excluded from the E2up active set^a^	84
Borderline active chemicals excluded from the P4up active set^a^	90
Chemicals single dose active response for E2	228
Chemicals single dose active response for P4	104
**Inactive**	
Inactive chemical set used for QSAR-E2up	1518
Inactive chemical set used for QSAR-P4up	1632

^a^Chemicals identified as active or borderline in H295R concentration–response by ANOVA as described in Cardona (2021).

Chemical structures were prepared following the best practices in the cheminformatics field, and chemicals without a ready structure were removed (see “Material and methods”)^25,26^. In total, 1701 structures were used to build the QSAR-E2up model with 10.7% of active chemicals, and 1813 chemicals with 10.3% actives were used for the QSAR-P4up model (Table 1). The lists of chemicals are available in Supplementary Information S1.

### E2up-P4up structural activity patterns

A structure-based SOM was computed on the full set of 1925 QSAR ready structures available from the 2021 chemicals in the H295R set. The optimal SOM was chosen with 64 clusters, with an average of 30 chemicals per cluster. An empirical approach was used to balance the number of chemicals by cluster and the number of empty clusters (Supporting Information Fig. S1). The selected SOM is presented in Supporting Information Fig. S2, colored by the number of chemicals by cluster. The smallest cluster (#57) included only four chemicals and the largest cluster (#42) included 67 chemicals.

**Figure 3:**
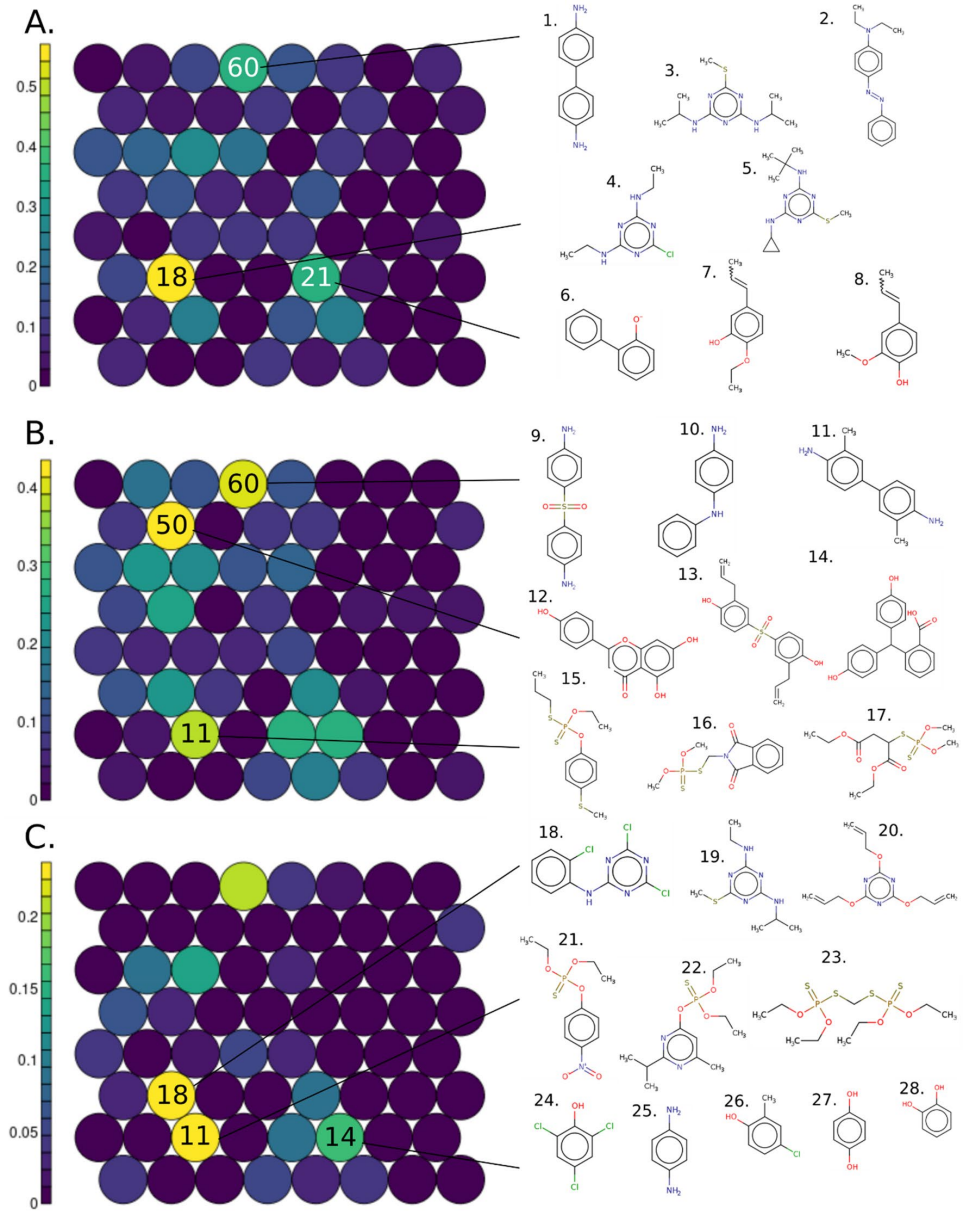
Structure based SOM on the 1,925 curated structures tested on the H295R assays including 64 clusters colored (**A**) using the percent of E2up chemicals, 182 structures, (**B**) using the percent of P4up chemicals, 186 chemicals and (**C**) the percent of the union of E2up and P4up structures, 71 structures. Some structural examples for enriched clusters are shown; cluster 60: 1. benzidine (92–87–5), 2. C.I. Solvent Yellow 56 (2481–94–9); cluster 18: 3. Prometryn (7287–19–6), 4. Simazine (122–34–9), 5. Cybutryne (28,159–98–0); cluster 21: 6. sodium 2-phenylphenate tetrahydrate (6152–33–6), 7. 2-ethoxy-5-(1-propenyl)phenol (94–86–0), 8. Isoeugenol (97–54–1); cluster 60: 9. Dapsone (80–08–0), 10. n-phenyl-1,4-benzenediamine (101–54–2), 11. 3,3′-dimethylbenzidine (119–93–7); cluster 50: 12. Apigenin (520–36–5), 13. 4,4′-dulfonylbis[2-(prop-2-en-1-yl)phenol] (41,481–66–7), 14. Phenolphthalin (81–90–3); cluster 11: 15. Sulprofos (35,400–43–2), 16. Phosmet (732–11–6), 17. Malathion (121–75–5); cluster 18: 18. Anilazine (101–05–3), 19. Ametryn (834–12–8), 20. 2,4,6-tris(allyloxy)-1,3,5-triazine (101–37–1); cluster 11: 21. Parathion (56–38–2), 22. Diazinon (333–41–5), 23. Ethion (563–12–2); cluster 14: 24. 2,4,6-trichlorophenol (88–06–2), 25. para-phenylenediamine (106–50-3), 26. 4-chloro-2-methylphenol (1570–64–5), 27. Hydroquinone (123–31–9) and 28. catechol (120–80–9). Please note that the color scales are different for each panel.

Next, the same SOM was colored based on cluster enrichment in E2up, P4up and E2up + P4up chemicals (Fig. 3). Enrichment was computed using the proportion (between 0 to 1) of each chemical set by cluster, and the most enriched clusters are highlighted.

Clusters 18, 21 and 60 show the most enrichment for E2up with 57%, 36% and 36% of E2up chemicals, respectively (Fig. 3a). Structurally, E2up chemicals in cluster 18 included triazine-amine ring compounds with sulfurous or chlorine groups. These substructures are prevalent in pesticides. For example, this cluster included simazine (4) and Prometryn (3) triazine herbicides. This cluster also included triazine derivative chemicals such as Cybutryne (5), used in paints to prevent biofouling. Some triazines are both P4up and E2up. For example, Anilazine (18) and Ametryn (19) are two herbicides that increase the production of E2 and P4 in the assay (Fig. 3c). Notably, some plastic-related substances such as the 2,4,6-tris(allyloxy)-1,3,5-triazine (20) are also found in this cluster.

Cluster 21 includes chemicals with phenol or benzenone derivative structures, (i.e., benzene group connected to a ketone or an alcohol group). Biphenyl chemicals are also found in this cluster, as for example, 2-phenylphenol (6), which are biocides often used as preservatives. This cluster also included some phenylpropenes such as isoeugenol (8) and 2-ethoxy-5-(1-propenyl)phenol (7) used as essential oils.

Cluster 60 is also enriched for E2up chemicals. Most of the chemicals in this cluster included an aromatic ring system with two benzene rings ramified with nitrogen groups such as primary, secondary or tertiary amine or diazene. Those chemical substructures are often used in dyes, such as benzidine (1) or C.I. Solvent Yellow 56 (2).

Cluster 60 was also enriched for P4up chemicals (39%), and thus it featured chemicals that are both E2up and P4up (Fig. 3). For example, Dapsone (9), which includes a phosphate group between two benzenes, was active for both hormones. Dapsone is a drug used for antibiotics and skin-care. Some dyes in cluster 60 were only P4up, such as aminodiphenylamine derivative (10) or dimethylbenzidine (11) (Fig. 3b).

Cluster 50 included 43% P4up chemicals. Structurally, these chemicals included phenol groups and complex aromatic systems ramified with oxygen-based groups such as flavone or phenolphthalein. These chemicals have varied uses including industrial, for example 4,4'-sulfonylbis[2-(prop-2-en-1-yl)phenol] (13) is used to produce resins, nutritional supplement for example Apigenin (12), or dye with for example phenolphthalein (14) that is used also as pH indicator.

Cluster 11 is also enriched for P4up activity, with 38% P4up chemicals. This cluster included chemicals with a dihydroxy phosphanedithione substructure. Notably, this cluster included numerous insecticides as Sulprofos (15), Phosmet (16) or Malathion (17). Many insecticides in this group also have both E2up and P4up activity (Fig. 3c), for example Parathion (21), Diazinon (22), and Ethion (23).

Finally, 17% of chemicals in cluster 14 were both E2up and P4up (Fig. 3c). Structurally, these chemicals are small, with only one benzene ramified with chlorine, alcohol, methyl or primary amine. For example, in this cluster we found 2,4,6-trichlorophenol (24), used as a pesticide, and catechol (28), widely used in pesticide production. This cluster also included drugs such as Hydroquinone (27), used as a depigmentation drug, and p-phenylenediamine (25) and 4-chloro-2-methylphenol (26), used for industrial dyes, polymer synthesis and drug manufacturing.

### Structural similarity between E2up and P4up chemicals and E2 and P4 hormones

**Figure 4:**
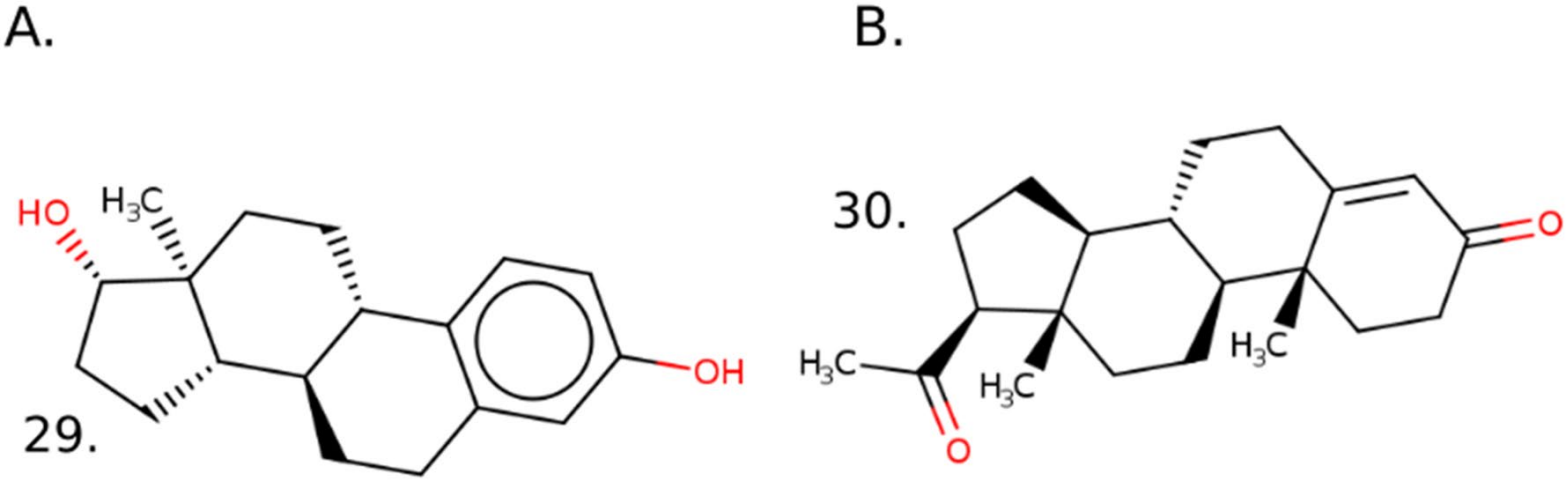
Chemical structure representation of (**A**) 29. Estradiol—E2 (DTXSID: DTXSID0020573, CASRN: 50–28–2) (**B**) and 30. Progesterone—P4 (DTXSID: DTXSID3022370, CASRN: 57–83–0).

E2 and P4 may influence steroidogenesis with positive or negative feedback controls, so we also examined whether active chemicals were structurally similar to E2 and P4. We found that most of the chemicals that increased E2 and P4 synthesis did not share a high similarity with endogenous hormones. Between them, the endogenous hormones E2 and P4 shared a similarity score of 0.58 using a MACSS fingerprint and Tanimoto metric. This high similarity is explained by the shared sterol derivative group (see structures in Fig. 4). Similarity was next computed between E2 and P4 and all of the chemicals of the H295R set with a QSAR ready structure and using the same method. Overall, the H295R set shared a similarity score of 0.25 ± 0.13 with E2 and 0.22 ± 0.12 for P4. E2up chemicals shared a similarity score of 0.27 ± 0.11 with E2 and 0.22 ± 0.10 with P4. P4up chemicals shared a similarity score of 0.27 ± 0.11 with E2 and 0.22 ± 0.12 for P4. Thus, E2up and P4up chemicals are not more structurally similar to E2 and P4 compared with the H295R set of tested chemicals.

**Figure 5:**
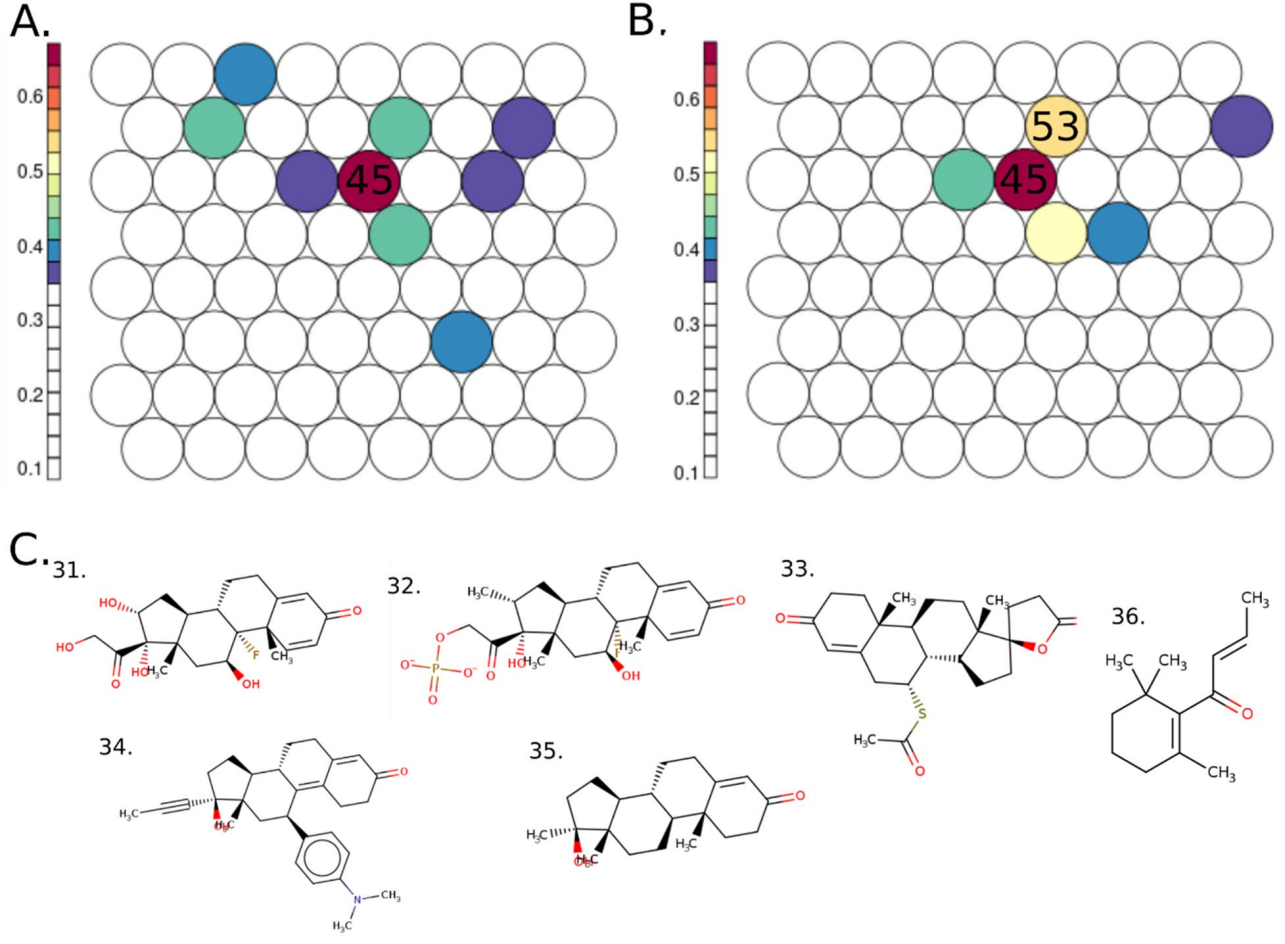
Structure based SOM on the H295R set including 64 clusters colored (**A**) using the average similarity score by cluster for E2 and (**B**) using the average similarity score by cluster for P4. Structures of E2up and P4up chemicals in clusters 45 and 53 are represented. Cluster 45: 31. Triamcinolone (124–94–7), 32. Dexamethasone sodium phosphate (2392–39–4), 33. Spironolactone (52–01–7), 34. Mifepristone (84,371–65–3) and 35. 17-methyltestosterone (58–18-4); cluster 53: 36. (E)-beta-damascone (23,726–91–2).

Two clusters in the SOM include chemicals with similar structures to E2 and P4, but these clusters were not enriched for chemicals that increased E2 or P4 steroidogenesis. The SOM in Fig. 5 is colored using the average similarity score by cluster for E2 and P4. Cluster 45 has the highest average similarity score for both hormones on average. Cluster 45 includes 35 chemicals and has an average similarity score equal to 0.67 for E2 and 0.68 for P4. For P4, cluster 53 included 13 chemicals with a high similarity with an average similarity score equal to 0.54. However, there is not enrichment in E2up and P4up chemicals in these clusters. Cluster 45 included only one E2up chemicals, the Triamcinolone (31) and five P4up including Triamcinolone (31), Dexamethasone sodium phosphate (32), Spironolactone (33), Mifepristone (34) and 17-Methyltestosterone (35). Structurally, all of them are drug and include a sterol derivative aromatic system which explain their high similarity with E2 or P4. Cluster 53 included only one P4up chemical, the (E)-beta-damascone (36) that is an essential oil used in perfumery as a fragrance. It is the major component of the rose aroma.

### Chemotypes for E2up and P4up chemicals

**Figure 6:**
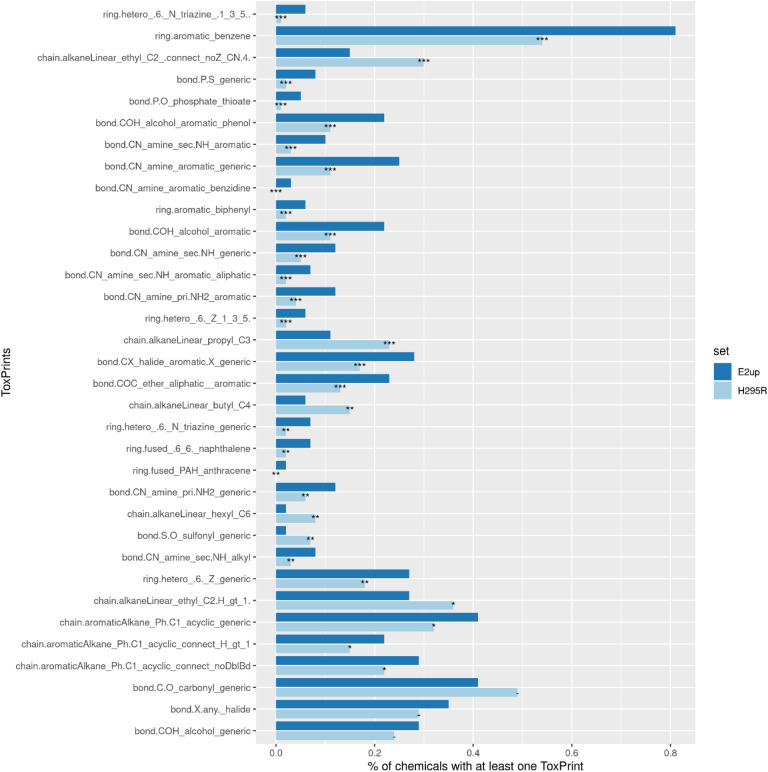
Distribution of ToxPrints that are significantly more common and the most represented (present in > 20% E2up chemicals) among the 729 ToxPrints identified in E2up chemicals. Statistical significance was computed using a Pearson's chi-squared test and significant p-values are reported as: p-value < 0.001 (***), p-value < 0.01 (**), p-value < 0.05 (*) and p-value ≥ 0.05 (−). ToxPrints are ordered by p-values, from the most significant to the least.

**Figure 7:**
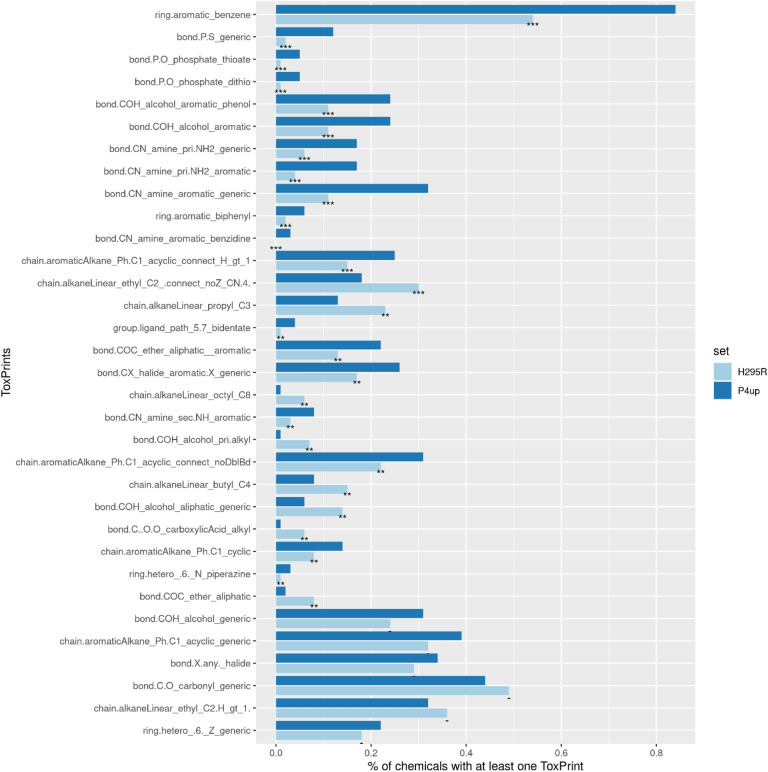
Distribution of ToxPrints that are significantly more common and the most represented (present in > 20% P4up chemicals) among the 729 ToxPrints identified in P4up chemicals. Statistical significance was computed using a Pearson's chi-squared test and significant p-values are reported as: p-value < 0.001 (***), p-value < 0.01 (**), p-value < 0.05 (*) and p-value ≥ 0.05 (−). ToxPrints are ordered by p-values, from the most significant to the least.

We computed ToxPrint chemotypes for each chemical in our E2up, P4up and total H295R sets. ToxPrints were specifically developed from a toxicity database and regulatory inventories to identify relevant chemotypes for toxicity prediction, as described in the “Material and methods” section and in Yuang et al.^29^. Each chemical in our dataset included on average 12 ToxPrints. We compared ToxPrints included in E2up and P4up sets against ToxPrints included in the H295R set of chemicals, Figs. 6 and 7. First, the most representative ToxPrint for E2up and P4up chemicals is the benzene ring ToxPrint (ring.aromatic_benzene) included in 81% of E2up chemicals, 84% of P4up chemicals and only in 54% of the H295Rset.

For both E2up or P4up chemicals, the most significant ToxPrints are amine derivative groups such as primary, secondary and tertiary amines included or not in an aromatic group (bond.CN_amine_sec.NH_generic, bond.CN_amine_pri.NH2_generic, bond.CN_amine_aromatic_generic, bond.CN_amine_sec.NH_aromatic, bond.CN_amine_pri.NH2_aromatic, bond.CN_amine_sec.NH_generic, bond.CN_amine_aromatic_benzidine). Phosphate group PO bonds (bond.P.O_phosphate_thioate, bond.P.S_generic) are also enriched in both the E2up or P4up sets.

For E2up chemicals specifically, the most significant ToxPrint is the ring included in the triazine structure (ring.hetero.6_N_triazine_.1_3_5). Eleven of the 19 chemicals that included this ToxPrint are E2up. This chemotype is not significant for P4up. Fused rings, such as in naphthalene and anthracene ToxPrints (ring.fused.6_6._naphthalene, ring.fused_PAH_anthracene), are also enriched for E2up chemicals. Three chemicals in the dataset included those structures and all of them are E2up. Finally, the sulfonyl group is underrepresented in the E2up chemicals, included in only 3 (2%) E2up chemicals compared with 140 (7%) in the full set of chemicals.

For P4up chemicals, ToxPrints that included a phosphate group (bond.P.O_phosphate_dithio, (bond.P.O_phosphate_thioate, bond.P.S_generic) are the most significantly enriched. Nine of the 11 chemicals that included a thioate are P4up.

**Figure 8:**
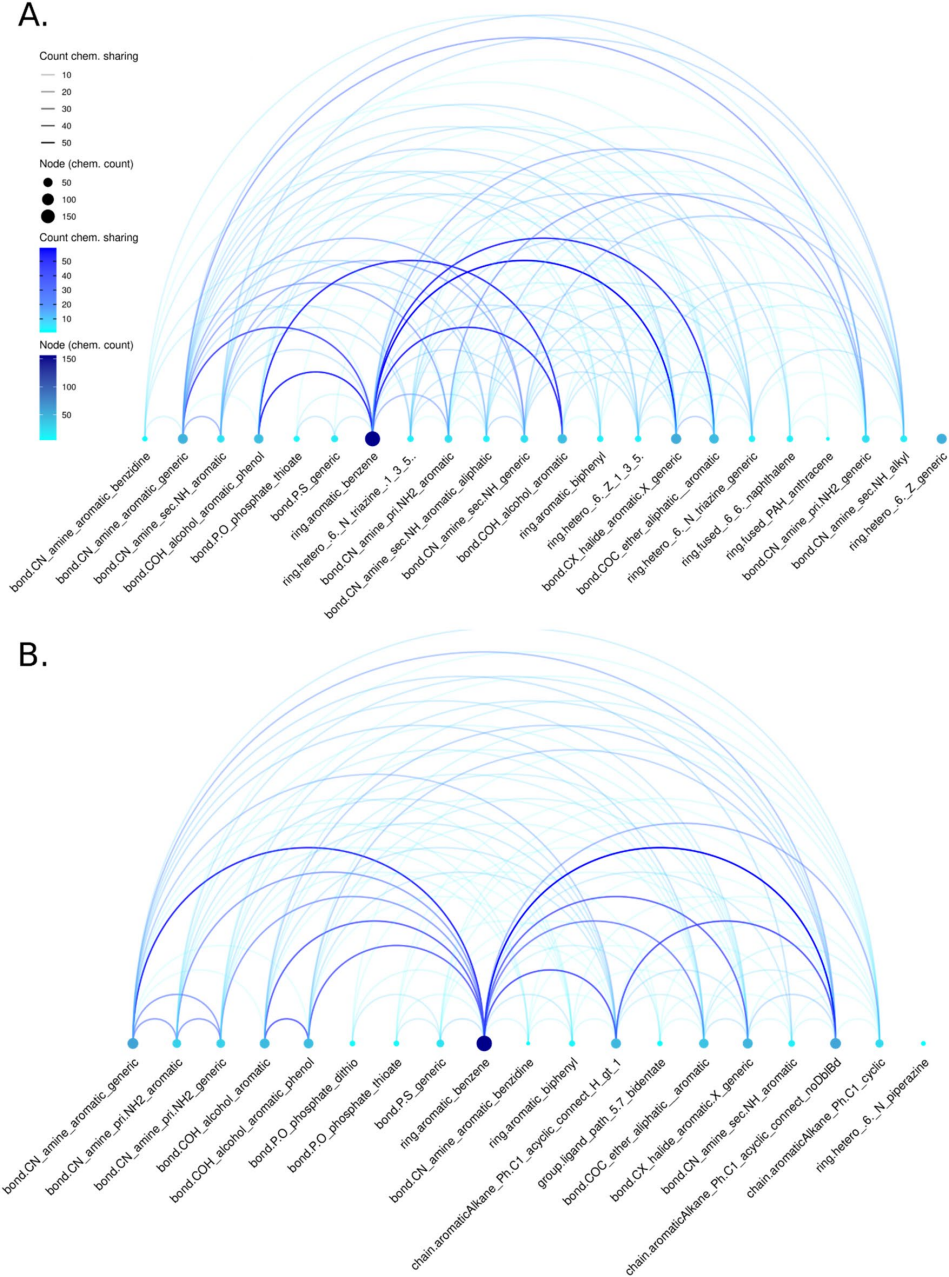
ToxPrint network for (**A**) E2up and (**B**) P4up chemicals. ToxPrints are represented by nodes colored by the number of the chemicals that included that ToxPrint. Combinations of ToxPrints are represented by the arc line colored based on the number of chemicals sharing these two ToxPrints. Only significant ToxPrints for E2up or P4up are represented (p-value < 0.01).

Next, we looked for common combinations of ToxPrints. On average, a chemical in the H295R set is composed of 12 ToxPrints, and E2up and P4up chemicals included on average three ToxPrints that were significantly enriched in E2up and P4up chemicals. However, ToxPrints characterize substructures, bonds and atoms, and we expect some dependency between them. For example, a phenol group includes an alcohol group, a benzene group, and some aromatic bonds, and so can be defined with a set of at least two ToxPrints (ring.aromatic_benzene, bond.COH_alcohol_aromatic_phenol). We analyzed combinations of chemotypes that are significant in E2up and P4up chemicals to refine the chemical profiling (Fig. 8). For both, ring_aromatics_benzene is the most represented ToxPrint with 146/186 E2up and 156/182 P4up chemicals including at least one benzene. Among the 146 E2up chemicals including benzene, 50 have a benzene connected to a halide group (bond.CX_halide_aromatic.X_generic), 48 have a phenol (bond.COH_alcohol_aromatic and bond.COH_alchool_aromatic_pheno) and 35 have an amine group connected to the benzene (bond.CN_amine_aromatic_generic). Among the 156 P4up chemicals that included a benzene, 58 included an benzene connected to an alkane chain (chain.aromaticAlkane_Ph.C1_acyclic_connect_noDblBd), 54 have a benzene with an amine (bond.CN_amine_aromatic_generic), 44 have a phenol group (bond.COH_alcohol_aromatic_pheno) and 44 included benzene with a halide group (bond.CX_halide_aromatic.X_generic).

This analysis highlights structural features that are enriched in E2- and P4up chemicals. Recognizing these features can aid in predicting whether any chemical might have this activity.

### QSAR modeling of E2up and P4up chemicals

To go further in structural modeling to predict E2up and P4up activity, we developed classification QSAR models using six different machine learning algorithms from a set of 1D and 2D structural descriptors combined with physicochemical properties. Two different models were developed—one for E2up chemicals (QSAR-E2up) and one for P4up chemicals (QSAR-P4up)—built using the E2up and P4up chemical sets as the active chemicals. Because only about 10% of the dataset was active for each activity, we applied an under sampling and non-under sampling approach with a GHOST method, as described in the “Material and methods”. Both approaches gave similar results, and only the non-under sampling approach is reported in Tables 2 and 3 because of less resources consuming. Each model was repeated five times with different data sampling between the training and the test set (test set = 15% of the set) and the best sampling was reported. In Supporting Information Table S1 we present the hyperparameters tested and chosen by machine learning with the optimization grid. All predictions were realized using a predicted probability score and the GHOST method was used to optimize performances in DNN and RF modeling^39^.

**Table 2:** Performance of QSAR classification models for E2up activity in H295R assay.

QSAR-E2up model (n structures = 1591)						
	Acc	b-Acc	Se	Sp	MCC	AUC
**Tenfold cross-validation (training set, n active = 153, n inactive = 1199)**						
CART	0.87	0.61	0.27	0.95	0.26	0.72
SVM-rbf	0.89	0.51	0.03	1	0.07	0.51
NN	0.89	0.5	0	1	0	0.73
LDA	0.87	0.64	0.34	0.94	0.31	0.78
DNN	0.78	0.64	0.45	0.82	0.21	0.64
RF	1	1	1	1	0.99	1
RF balanced	0.77	0.86	0.98	0.75	0.49	0.86
**Fitting (training set, n active = 153, n inactive = 1199)**						
CART	0.96	0.83	0.67	1	0.78	0.88
SVM-rbf	1	1	1	1	1	1
NN	0.92	0.68	0.37	0.99	0.52	0.68
LDA	0.91	0.72	0.47	0.97	0.51	0.91
DNN	0.86	0.69	0.47	0.9	0.34	0.69
RF	1	1	1	1	0.99	1
RF balanced	0.77	0.86	0.98	0.75	0.49	0.86
**External validation (test set, n active = 27, n inactive = 212)**						
CART	0.86	0.65	0.37	0.92	0.29	0.74
SVM-rbf	0.89	0.5	0	1	0	0.5
NN	0.88	0.58	0.19	0.97	0.24	0.58
LDA	0.84	0.6	0.3	0.91	0.21	0.77
DNN	0.84	0.71	0.56	0.87	0.36	0.71
RF	0.9	0.72	0.48	0.96	0.48	0.72
RF balanced	0.76	0.83	0.93	0.74	0.45	0.83

*Acc* accuracy, *b-Acc* balanced accuracy, *Sp* specificity, *Se* sensitivity, *AUC* Area Under the Receiver Operating Characteristic Curve, *MCC* Matthew Coefficient Correlation, see “Material and methods”.

**Table 3:** Performance of QSAR classification models for P4up activity in H295R assay.

QSAR-P4up model (n structures = 1700)						
	Acc	bAcc	Se	Sp	MCC	AUC
**Tenfold cross-validation (training set, n active = 155, n inactive = 1290)**						
CART	0.87	0.61	0.29	0.94	0.25	0.69
SVM-rbf	0.89	0.52	0.04	1	0.11	0.52
NN	0.89	0.5	0	1	0	0.71
LDA	0.88	0.62	0.3	0.95	0.28	0.76
DNN	0.83	0.62	0.36	0.89	0.24	0.62
RF	0.99	0.99	0.98	0.99	0.95	0.99
RF balanced	0.76	0.86	1	0.73	0.47	0.86
**Fitting (training set, n active = 155, n inactive = 1290)**						
CART	0.97	0.87	0.75	1	0.83	0.92
SVM-rbf	0.99	0.97	0.95	1	0.97	0.97
NN	1	0.99	0.99	1	0.99	0.99
LDA	0.91	0.71	0.44	0.97	0.49	0.92
DNN	0.52	0.71	0.97	0.46	0.27	0.71
RF	0.99	0.99	0.98	0.99	0.94	0.99
RF balanced	0.76	0.86	1	0.73	0.47	0.86
**External validation (test set, n active = 26, n inactive = 229)**						
CART	0.89	0.66	0.37	0.95	0.35	0.78
SVM-rbf	0.9	0.52	0.04	1	0.18	0.52
NN	0.91	0.56	0.11	1	0.32	0.56
LDA	0.87	0.58	0.22	0.94	0.19	0.76
DNN	0.5	0.67	0.45	0.89	0.21	0.67
RF	0.89	0.66	0.37	0.96	0.37	0.66
RF balanced	0.73	0.8	0.89	0.71	0.39	0.8

*Acc* accuracy, *bAcc* balanced accuracy, *Sp* specificity, *Se* sensitivity, *AUC* Area Under the Receiver Operating Characteristic Curve, *MCC* Matthew Coefficient Correlation, see “Material and methods”.

Globally, QSAR-E2up models performed better than QSAR-P4up models despite a similar number of chemicals and similar ratio between active and inactive chemicals. The best MCC on the test set for the QSAR-E2up model is 0.45 and 0.39 for the QSAR-P4up model with a balanced RF algorithm. For both the E2up and the P4up models, decision tree (CART), SVM with a rbf kernel and neural network singleton (NN) models are the weakest models with an MCC in cross validation that is less than 0.26. Low performances can be explained by poor sensitivity, i.e., the inability to predict true positives.

More advanced machine learning algorithms including DNN and RFs generally performed better on this dataset. This is probably because of the unbalanced sets, with more inactive than active chemicals, which make it more difficult to model with classic machine learning. Performance was also improved by the GHOST optimization used for those algorithms. For the QSAR-E2up models, RF performs better than DNN with an MCC on the test set equal to 0.48 and 0.36, respectively. For QSAR-P4up the same tendency is found with an MCC on the test equal to 037 for RF and 0.21 for DNN. Again, for both QSAR models, the low performance is explained by a weak sensitivity, with a sensitivity of 0.56 for DNN and 0.48 for RF for QSAR-E2up test set and 0.45 and 0.35, respectively, for QSAR-P4up test set.

Of all the approaches, the best predictions were observed using the balanced RF algorithm, since they had the most balanced sensitivity and specificity. This algorithm exhibits the best-balanced accuracy for QSAR-E2up and QSAR-P4up, equal to 0.86 and 0.86 on the respective cross validation and 0.83 and 0.80 on the respective test sets. Other machine learning algorithms do not exceed 0.72 in terms of balanced accuracy with a sensitivity always below 0.56 in cross validation and on the test set.

**Figure 9:**
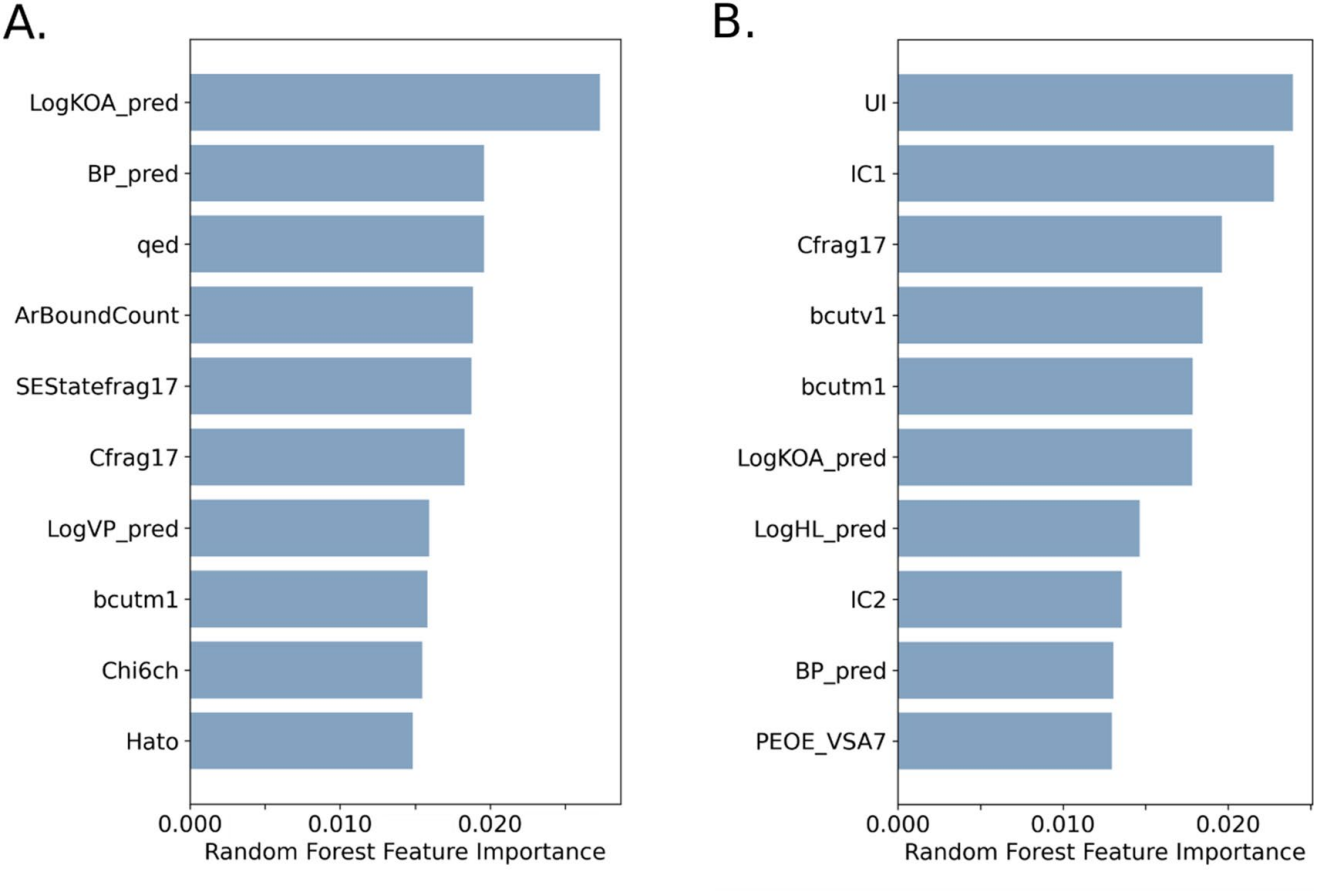
Top 10 of descriptor importance, in relative value, of features for balanced RF developed for (**A**) E2up and (**B**) P4up QSAR models.

The top 10 important descriptors involved in the balanced QSAR-E2up and QSAR-P4up RF models are presented in Fig. 9 and Table 4. Descriptors correlated to aromatic substructures play an important role in both models. For QSAR-E2up, the count of aromatic bonds (ArBondCount) is one of the top 10 descriptors. On average, E2up and P4up chemicals have 9 and 10 aromatic bonds, respectively, which is significantly higher than chemicals in the H295R set that included only 6 aromatic bonds on average. In addition, in both models the count of the aromatic fragment frag17 (aasC: two aromatic bonds connected to one single bond and a carbon) is present in the top 10 with a higher count for E2up (3.14) and P4up (3.40) than for the H295R set (2.01). For the QSAR-E2up model specifically, the sum of the electrotopological state (E-states) for the same fragment 17 (descriptor SEStatefrag17) is also one of the top 10 descriptors. For QSAR-P4up, the unsaturated index (UI), directly connected to the number of aromatic rings, is found in the top 10. For both of these descriptors, their average values in the E2up set or in P4up set are higher than their average values in the H295R set. For SEStatefrag17, the average value in the E2up chemicals set is 1.56 *versus* 0.92 in the H295R set. For UI, the average value in the P4up is 3.45 *versus* 2.54 in the H295R set. For the QSAR-E2up model, aromatic composition is also found with the Chi connectivity index of cycle of 6 (Chi6ch), and the average value of this descriptor is also significantly higher in the P4up set than in the full set of chemicals (0.13 *versus* 0.09).

**Table 4:** Details of the Top 10 descriptors from the best RF models, including average (M) of the descriptor values for each group of E2up, P4up and H295R, with an associated p-value significance (*** < 0.005).

Descriptors	M E2up	M P4up	M H295R
**MOE-type descriptors**			
PEOE_VSA7 MOE-type descriptors using partial charges and surface area contributions	31.67 ± 16.38***	33.80 ± 18.35***	24.35 ± 20.22
SEStatefrag17 E-states for the fragment aasC (two aromatic bonds, one single bond and a carbon)	1.56 ± 1.76***	1.70 ± 1.96***	0.92 ± 1.72
**Physicochemical descriptors**			
LogVP_pred Log Vapor pressure prediction (degree Celsius)	− 5.72 ± 2.51***	− 6.06 ± 2.57***	− 4.44 ± 3.33
LogKOA_pred Log of octanol/air partition coefficient prediction	8.95 ± 1.45***	9.18 ± 1.58***	7.80 ± 2.38
LogHL_pred Log of Henry’s Law constant (air/water partition coefficient) at 25C prediction	− 7.26 ± 1.57**	− 7.56 ± 1.50***	− 6.76 ± 2.20
BP_pred Boiling point prediction	328.23 ± 53.09***	334.25 ± 58.50***	285.70 ± 80.64
**Molecular property descriptor**			
qed quantitative estimation of drug-likeness	0.63 ± 0.14***	0.61 ± 0.14***	0.54 ± 0.16
UI Unsaturation index	3.31 ± 0.86***	3.45 ± 0.71***	2.54 ± 1.34
**Topological descriptor**			
Hato Harmonic topological index proposed by Narnumi	1.81 ± 1.68***	1.78 ± 0.158***	1.70 ± 0.20
**Composition**			
ArBoundCount Count of aromatic bound	9.42 ± 5.52***	9.90 ± 5.83***	6.0 ± 6.03
Cfrag17 Count of fragment aasC (two aromatic bonds, one single bond and a carbon)	3.14 ± 1.89***	3.37 ± 1.99***	2.01 ± 2.22
**Basak descriptors**			
IC1 “Steric hindrance” around a path of order 1	2.61 ± 0.48***	2.70 ± 0.39***	2.52 ± 0.55
IC2 “Steric hindrance” around a path of order 2	3.39 ± 0.62***	3.50 ± 0.58***	3.11 ± 0.75
**Bcut**			
Bcutm1 Lowest eigen value 1 of Burden matrix/weighted by atomic mass	3.68 ± 0.44***	3.72 ± 0.51***	3.56 ± 0.65
Bcutv1 Lowest eigen value 1 of Burden matrix/weighted by vander Waals volumes	3.87 ± 0.12***	3.89 ± 0.11***	3.78 ± 0.20
**Connectivity**			
Chi6ch Simple molecular connectivity Chi indices for cycles of 6	0.13 ± 0.08***	0.13 ± 0.08***	0.09 ± 0.09

Student-test or Wilcoxon test (if descriptor distribution was not normal) were applied.

Physicochemical properties are influential in the QSAR-E2up and QSAR-P4up models, with four physicochemical descriptors in the top 10 most important descriptors: vapor point (LogVP_pred), the octanol/air partition coefficient (LogKOA_pred), the boiling point (BP_pred) and the Henry’s Law constant (LogHL_pred). For all of these descriptors, there is a significant difference between their values in the E2up and P4up chemical sets and the H295R set.

Charge distribution around the chemicals is also an important property in the top 10 important descriptors for QSAR-P4up. The MOE partial charge surface area contributions descriptors PEOE_VSA7 is found higher on average in E2up and P4up sets than in the H295R full set.

Finally, other descriptors that characterized the connectivity, topology and chemical shape, complete the top 10 important descriptors for both models. For QSAR-E2up, these include the quantitative estimation of drug-likeness (qed) the harmonic topological index proposed by Narnumi (hato) and the burden descriptors of index 1 weighted by the atomic mass (bcutm1). For the QSAR-P4up model, they include two basak descriptors (IC1, IC2) and two burden descriptors, both related to the first chemicals eigen value weighted by the atomic mass (bcutm1) and the van der Waals volumes (bcutv1).

Globally, all of these descriptors included in the top 10 have significantly different values between E2up, P4up chemicals and the H295R chemical set.

### Predicting steroidogenic activity of mammary carcinogens

**Figure 10:**
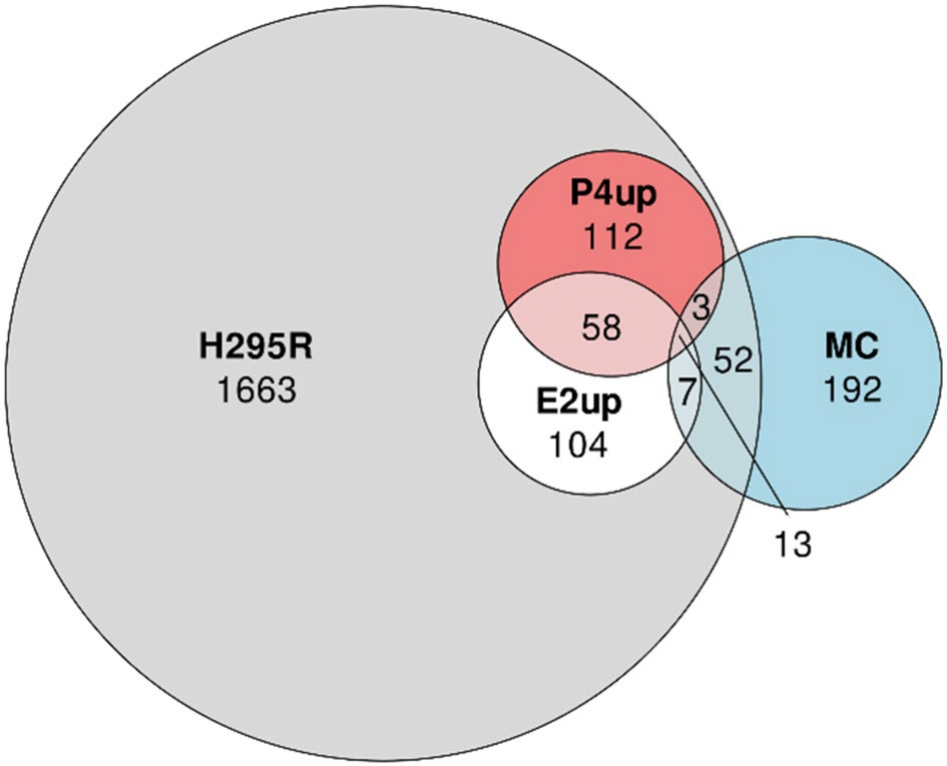
Venn diagram between active E2up, P4up, H295R and MC chemical sets.

Finally, we applied the QSAR-E2up and QSAR-P4up models to a set of 267 mammary carcinogens (MC) compiled from in vivo evidence of carcinogenicity. Our goal was to predict their ability to increase steroidogenesis, since we hypothesize that this is an important mechanism for mammary carcinogenesis. Figure 10 presents the overlap between the MC set with the E2up, P4up and H295R chemical sets. Among the full set of MCs, only 75 were tested on the H295R assay, with 45 in CR. This count included 5 hormones or subtracts. Twenty-three are actives, including 13 actives for both E2up and P4up, 7 only active in E2up, and 3 only in P4up. Among the 75 MCs tested on H295R, 52 are inactive for both production of E2 and P4. Finally, 194 chemicals have not been tested on the H295R. To prioritize them for future testing, we applied our QSAR models on this set.

**Figure 11:**
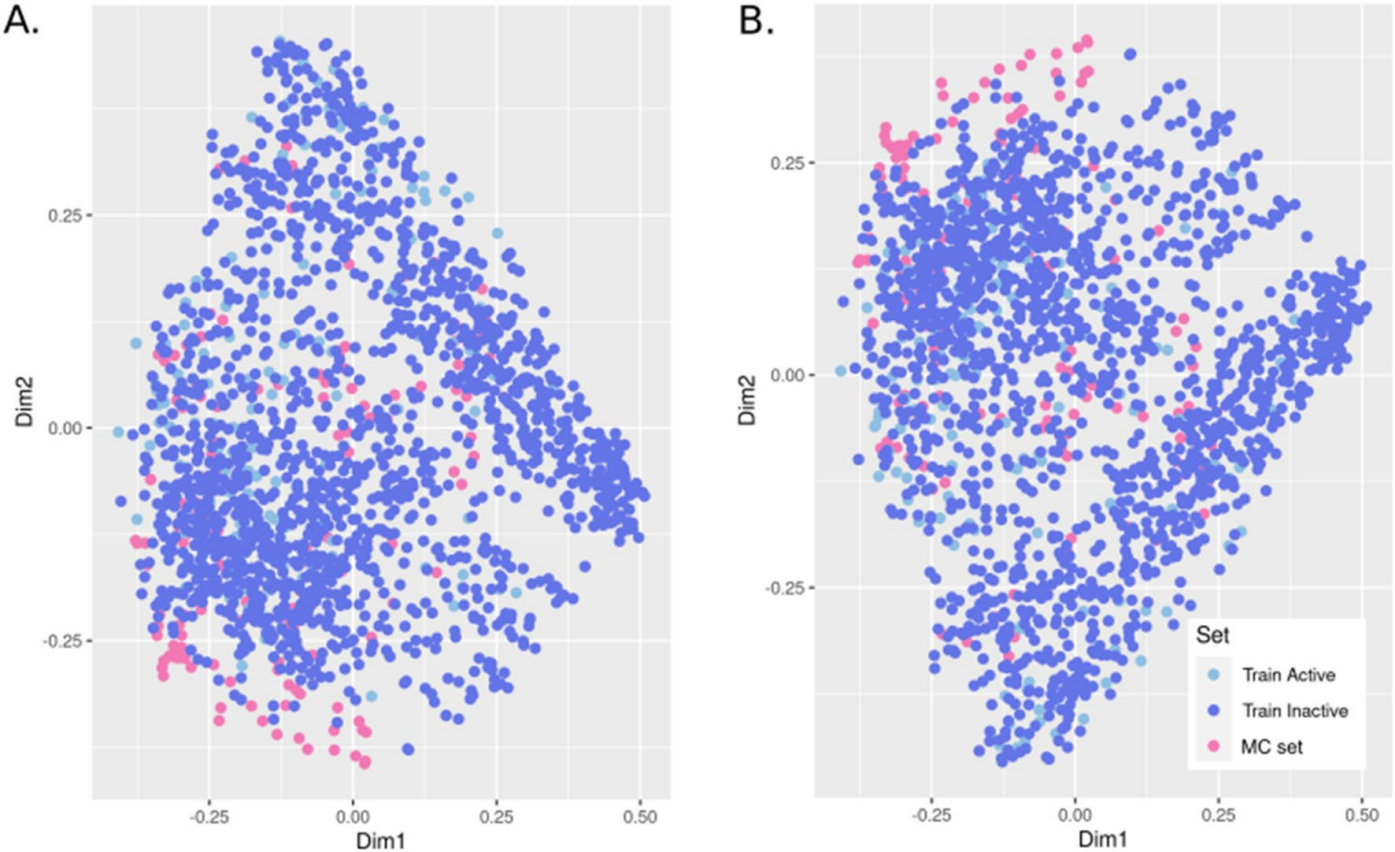
Projection of the MC chemicals set on the training set used to build the (**A**) QSAR-E2up model and (**B**) QSAR-P4up model. Projection is realized using a multidimensional scaling on the similarity matrix computed using a pair wise Tanimoto score from a MACSS fingerprint by chemicals.

The list of MC chemicals was developed using several resources that give in vivo evidence that these chemicals cause mammary gland tumors. This set includes a diverse set of chemical structures. First, we reviewed QSAR model applicability domains for the MC list. Figure 11 shows the projections of the similarity matrix, based on MACCS fingerprint and a Tanimoto score, of the training set combined with the MC chemicals. We found that many of the MCs were located in the extremity of the projection, which shows a poor similarity with chemicals in the training set. To make sure we only applied the QSAR model to chemicals within the relevant AD, we computed an AD score for each prediction that is equal to the minimum Tanimoto score between the chemical and chemicals in the training set.

**Figure 12:**
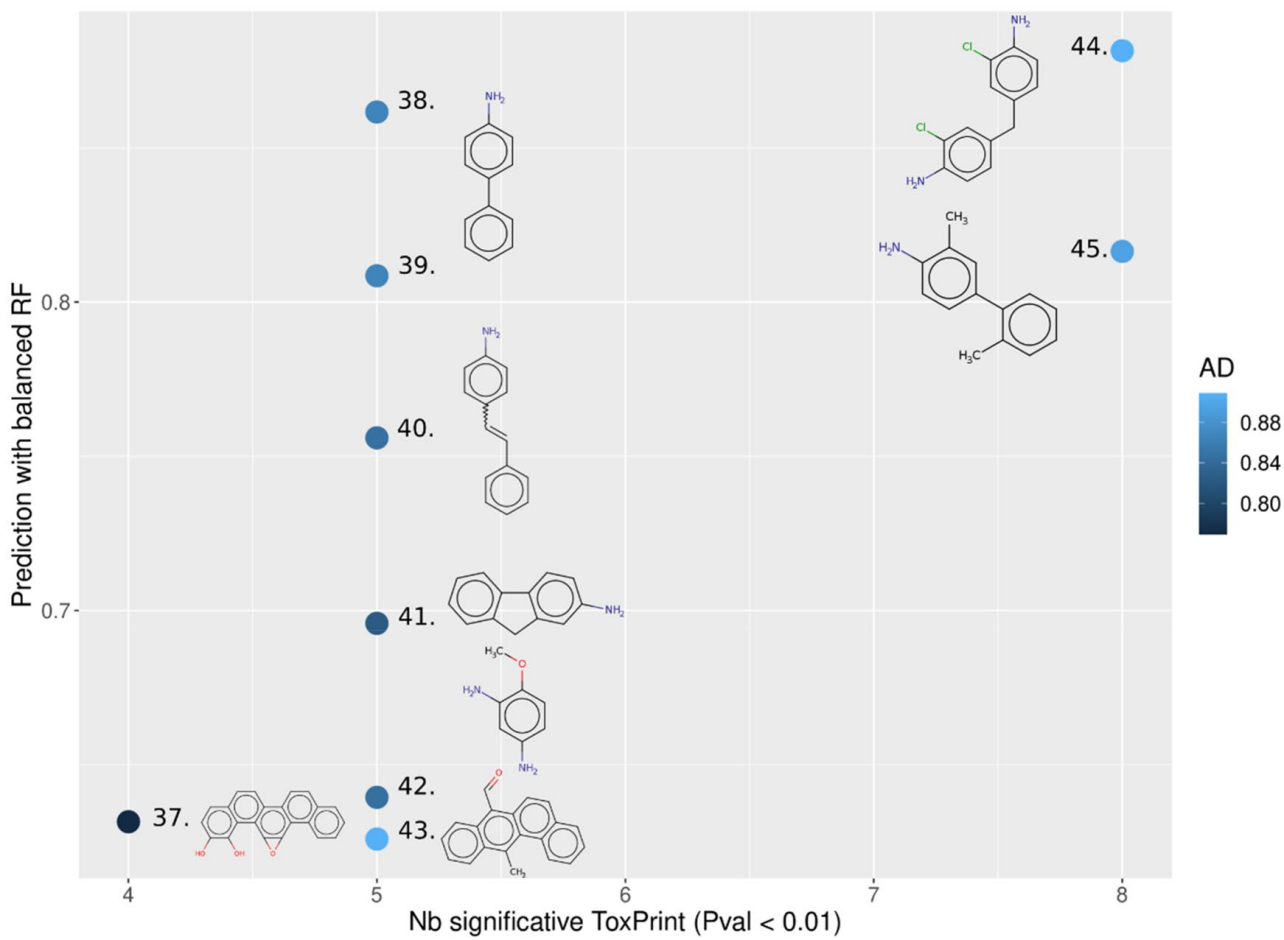
Predicted E2up chemicals from the MC set of chemicals. The x-axis represents the number of ToxPrints significant for E2up, the y-axis represents the probability prediction to be E2up, and the applicability model is in blue (minimal similarity score with the first chemicals in the training set). Chemical structures are represented in the figure: 37. anti-benzo[a]chrysene-11,12-diol-13,14-epoxide (132,832–26–9), 38. 4-biphenylamine (92–67–1), 39. p-aminobiphenyl hydrochloride (2113–61–3), 40. 4-aminostilbene (834–24–2), 41. 2-aminofluorene (153–78–6), 42. 2,4-diaminoanisole sulfate (39,156–41-7), 43. 12-methylbenz(a)anthracene-7-carboxaldehyde (13,345–61–4), 44. 4,4′-methylenebis(2-chloroaniline) (101–14–4) and 45. 3,2′-dimethyl-4-aminobiphenyl (13,394–86–0).

MCs that are predicted to be E2up and P4up with the highest confidence are shown in Fig. 12. These chemicals had a probability to be E2up or P4up above 0.5, had an AD score above 0.75, and included more than three significant ToxPrint chemotypes. The full list of MCs with their predicted probability of being E2up or P4up using these QSARs is included in Supplementary Information S2. We found that only 9 and 15 chemicals are predicted active with a high confidence with QSAR-E2up and QSAR-P4up models, respectively. Next, we found 33 and 36 chemicals predicted E2up or P4up with a medium confidence, and 24 and 27 with a low confidence, using QSAR-E2up and QSAR-P4up, respectively.

Among the nine chemicals predicted E2up with a high confidence, five are also predicted to be P4up: anti-benzo[a]chrysene-11,12-diol-13,14-epoxide (37), 4-biphenylamine (38), p-aminobiphenyl hydrochloride (39), 4-aminostilbene (40) and the 4,4′-methylenebis(2-chloroaniline) (44). Finding chemicals that are active for both models is consistent with the training set that included 71 chemicals that are active for both. We found similar substructures as were discussed in the clustering step—predicted active chemicals included primary amine or chlorine groups ramified on a benzene, for example in chemicals 38, 39, 40 and 44 (Fig. 12).

**Figure 13:**
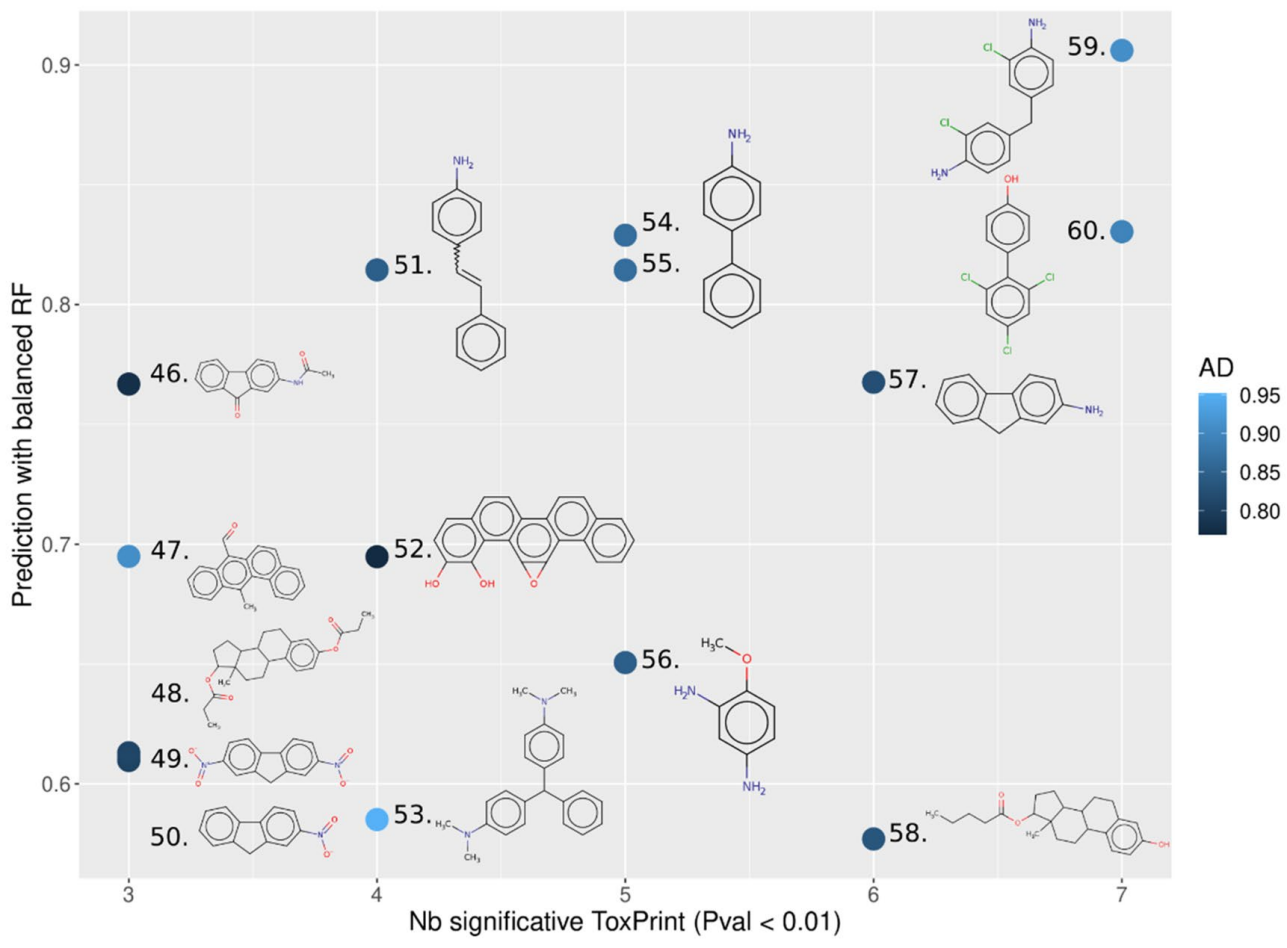
Predicted P4up chemicals from the MC set of chemicals. The x-axis represents the number of ToxPrints significant for P4up, the y-axis represents the probability prediction to be P4up, and the applicability model is in blue (minimal similarity score with the first chemicals in the training set). Chemicals structural are represented in the figure: 46. n-(9-oxo-2-fluorenyl)acetamide (3096–50–2), 47. 12-methylbenz(a)anthracene-7-carboxaldehyde (13,345–61–4), 48. estradiol dipropionate (113–38–2), 49. 2,7-dinitrofluorene (5405–53–8), 50. 2-nitrofluorene (607–57–8), 51. 4-aminostilbene (834–24–2), 52. anti-benzo[a]chrysene-11,12-diol-13,14-epoxide (132,832–26–9), 53. Leucomalachite green (129–73–7), 54. 4-biphenylamine (92–67–1), 55. p-aminobiphenyl hydrochloride (2113–61–3), 56. 2,4-diaminoanisole sulfate (39,156–41–7), 57. 2-aminofluorene (153–78–6), 58. estradiol valerate (979–32–8), 59. 4,4′-methylenebis(2-chloroaniline) (101–14–4) and 60. 3,2′-dimethyl-4-aminobiphenyl (13,394–86–0).

For both models, these predictions confirmed that active chemicals are enriched in aromatic substructures, including, for example, benzene groups or more complex aromatics substructures such as dibenzophenanthrene (37, 34, 47 and 52) or fluorene (41, 46, 49, 50 and 57) (Figs. 12 and 13).

We attempted to use a PubMed literature search to confirm the predicted increase in synthesis of E2 or P4, however, there were few articles that reported relevant endpoints. We searched the 17 chemicals that were predicted with high confidence to be E2up or P4up, using key words related to steroidogenesis or E2 and P4 levels (see “Material and methods”). We reviewed article titles from PubMed for 8 QSAR-E2up and 15 QSAR-P4up chemicals looking for publications that might report an outcome of altered serum or tissue E2 or P4 or a closely related effect. No articles were found for 4/8 predicted E2up and 9/15 predicted P4up chemicals. Two E2-related chemicals that were predicted P4 up had many publications because they are used as hormone therapy for menopausal symptoms (estradiol dipropionate, n = 52; estradiol valerate, n = 27,012), so we reviewed only the top 20 titles sorted by best match to the search query, but we did not find papers relevant to how these treatments influenced P4 production. Based on the titles of articles returned for the other chemicals in the search, we found one article relevant to the chemical 4-biphenylamine and its salt. Kumar et al.^40^ report that 4-biphenylamine increases activity of three enzymes involved in steroidogenesis: CYP11A1, 3β-HSD and 17β-HSD. The study measured increased production of androgens, but did not measure E2 or P4 levels (our QSARs predict that 4-biphenylamine would increase both). However, review of the steroidogenic pathway suggests that increasing activity of these three enzymes would likely also increase E2 and P4 because these enzymes produce them. Thus, we found some confirmation that 4-biphenylamine increases activity of three enzymes in the steroidogenic pathway, but other than that, few studies were available that examined relevant endpoints. This result indicates an overall sparse knowledge of the potentially related effects of the chemical, especially on the steroidogenesis pathway.

Thus, in general the literature review did not produce useful information to validate or invalidate the QSAR predictions.

## Discussion

In this work we provide a cheminformatics analysis of chemicals that increase production of E2 and P4^5^ in an internationally validated in vitro assay.

First, we analyzed E2up and P4up chemicals using structural clustering and chemotypes. We did not find structural similarity between E2 or P4 and chemicals that increase synthesis of E2 or P4.

This structural analysis can guide flagging of structural alerts in screening programs. It can also help to pinpoint potential mechanisms of action for altered steroidogenesis, for example by generating hypotheses about target structures that can impact the steroidogenesis pathway. The large structural diversity of chemicals that are E2up or P4up suggests that many modes of action are involved in the production of E2 and P4. Potential modes of action might include activation of enzymes in the synthetic pathway^41^, altered transcription to regulate enzyme production^42,43^, or others^15,20^. Finally, understanding structural features of these E2up and P4up chemicals can provide insights into important biological pathways that influence breast cancer progression, given the importance of these pathways for breast cancer.

We also built classification QSAR models to predict if a chemical is likely to increase E2 or P4 steroidogenesis based on its structure. Some RF balanced models with a GHOST procedure demonstrated reasonable performance, with balanced accuracy on the test set equal to 0.86 and 0.80 with an associated MCC equal to 0.49 and 0.39 for the QSAR-E2up and QSAR-P4up models, respectively, in tenfold cross validation. One limitation of the performance can be explained by the small set of active structures, excluding chemicals that we can’t prepare for QSAR modeling (only 153 and 155 active chemical structures for QSAR-E2up and QSAR-P4up) and the imbalance between the number of active and inactive chemicals that limit a good coverage of the active chemical space. This limitation has been reported with other in silico prediction efforts^44,45^. To strengthen the prediction from our QSARs, we also used structural information related to applicability domain and ToxPrints to build a confidence index score. These models can be used to identify chemicals that are likely to increase E2 or P4 steroidogenesis from large sets of chemicals in a computational high throughput screening effort.

Because of the strong evidence linking E2 and P4 action with breast cancer, we applied the QSARs to a set of 194 chemicals shown to induce mammary tumors in rodents but not tested in H295R. We hypothesized that this set of chemicals would be enriched in E2- and P4up activity because we found that MCs tested in H295R were enriched for this activity^5^. Our list of MCs is an update of the rodent mammary carcinogen list we published in 2007^23^. After prefiltering, QSAR-E2up and QSAR-P4up models were applied on this set of chemicals. We found 53 and 66 chemicals predicted to be active for increasing either E2 or P4 synthesis, however only 8 and 15 chemicals are predicted with high confidence based on having a structure within the applicability domain of the QSAR models. In fact, of the 267 rodent MCs, only 75 were tested in the H295R assay, which is surprising given the relevance of the pathway to breast cancer. The H295R assay is an internationally validated assay for steroidogenesis activation for regulation policy. We expect that more in vitro testing and public release of the data can extend the applicability domain of in silico models.

The literature review we conducted to try to confirm steroidogenic effects of the predicted E2- and P4up chemicals produced very little relevant information to validate or invalidate the QSAR predictions because relevant endpoints (increased production of E2 and P4) have not been studied. However, 4-biphenylamine, which is predicted by the QSAR to increase E2 and P4, had one study showing increased gene expression of three steroidogenic enzymes (cytochrome P450scc, 3β-hydroxysteroid dehydrogenase, 17β-hydroxysteroid dehydrogenase) that could result in higher levels of these hormones^40^.

As discussed above, evidence from several domains provides strong support for the hypothesis that chemicals that increase estradiol and progesterone levels or ER activation likely increase breast cancer risk. In both rats and mice, hormone supplementation increases mammary tumors, and in humans, ovariectomy reduces breast cancer risk while hormone replacement therapy increases it^4^. ER antagonists and aromatase inhibitors are first line therapies to prevent breast cancer recurrence in women with hormone receptor positive breast cancers. However additional studies are needed to determine how the modes of action that are captured in the H295R in vitro steroidogenesis assay operate in vivo, and whether effects are modified by polymorphisms in steroidogenic enzymes^46,47^ and feedback inhibition, for example. Another priority question is to discern effects of chemicals on systemic (ovarian, liver) vs. local hormone production in relation to breast carcinogenesis and other health outcomes that may result from increased production of estradiol and progesterone^46,48^.

Chemicals that increase E2 and P4 steroidogenesis are likely to increase breast cancer risk and affect other breast-related endpoints, such as breast development and lactation^5,49–51^. Thus, QSAR models and structural alerts that predict these chemicals can play an important role in flagging chemicals of concern for further study and exposure reduction. The link between the E2 and P4 and breast cancer is already well established, and most treatments to reduce risk of recurrence block these pathways. Historically, more attention has been focused on how c ER-active chemicals may affect breast cancer^1,52,53^, while the potentially important role of chemical steroidogens has only recently been highlighted^5^. Additional work is needed to develop methods for measuring effects on steroidogenesis, to collect that data in vivo, and to characterize the consequences of exposure to chemicals with these steroidogenic effects.

## Supplementary Information

Supplementary Information 1.

Supplementary Information 2.

Supplementary Information 3.

Supplementary Information 4.

## Data Availability

All data generated or analyzed or scripts developed during this study are included in this article or are included in a GitHub repository available at https://github.com/SilentSpringInstitute/StructuralAnalysisE2upP4up.
