# Supplementary material for: Cheminformatics analysis of chemicals that increase estrogen and progesterone synthesis for a breast cancer hazard assessment
Source: Sci Rep. 2022 Nov 30;12:20647. doi: 10.1038/s41598-022-24889-w (PMC9712655; doi:10.1038/s41598-022-24889-w)
Supplement: Supplementary file 1 — Supplementary Information 1. [file 41598_2022_24889_MOESM1_ESM.docx]

Supporting Information: Cheminformatics analysis of chemicals that increase estrogen and progesterone synthesis for a breast cancer hazard assessment

Alexandre Borrel* & Ruthann Rudel

Silent Spring Institute, Newton, Massachusetts, USA

Contact: [borrel@silentspring.org](mailto:borrel@silentspring.org)

**Supporting Information S1:** excel file including list of chemicals used for this study.

**Supporting Information S2:** excel file including QSAR-E2up and QSAR-P4up prediction for the mammary carcinogen list.

**Supporting Information S3:** excel file including PubMed search results for MC chemicals predicted E2up and P4up.

**Table S1**: Parameters and hyperparameters screened for each machine learning developed. When parameters are not reported the default is kept.

| Machine learning | Grid optimization methods and/or parameters screened | Parameter selected | |
| --- | --- | --- | --- |
|  |  | QSAR-E2up | QSAR-P4up |
| CART  (R 4.1 – rpart lib) | *rpart.control* function | *minsplot*:1  *maxsplit*: 30 | *minsplot*:1  *maxsplit*: 30 |
| NN  (R 4.1 – nnet lib) | *nnet* method  maxit: 75  *decay*: [0.1, 0.5, 1]  *vsize*: [1, 2, 5] | *vsize*: 1  *decay*: 5 | *vsize*: 0.1  *decay*: 5 |
| DNN (Python 3.9 - tensorflow) | *kernel_inirialization*: random normal  *optimizer*: adam  *activation*: [relu, selu]  *epochs*: [50, 100, 120]  *batch_size*: [32, 64, 128]  *dense_layer*: [3, 4, 5]  *dense_candidate*: [50, 25, 20, 10, 1]  *GHOST criteria:* [0:1,0.05] | *activation*: selu  *epochs*: 50  *batch_size*: 32  *dense_layer*: 3  *dense_candidate*: 50  *GHOST criteria*: 0.4 | *activation*: selu  *epochs*: 50  *batch_size*: 32  *dense_layer*: 3  *dense_candidate*: 50  *GHOST criteria*: 0.35 |
| SVM- kernel rbf  (Python 3.9 sklearn lib) | *vcost:* [0.1, 1, 2, 10, 100]  *vgamma:* [1, 0.1, 0.01, 0.001, 0.0001] | *vcost:* 2  *vgamma:* 0.001 | *vcost:* 2  *vgamma:* 0.001 |
| RF  (Python 3.9 sklearn lib) | *n_estimators :* [10, 50, 100, 300, 500, 800, 1200]  *max_depth:* [5, 8, 15, 25, 30]  *min_samples_leaf*: [1, 2, 5, 10]  *min_samples_split*: [2, 5, 10, 15, 100]  *GHOST criteria:* [0:1,0.05] | *n_estimators :* 1200  *max_depth:* 15  *min_samples_leaf*: 2  *min_samples_split*: 5  *GHOST criteria:* 0.35 | *n_estimators : 500*  *max_depth:* 15  *min_samples_leaf*: 5  *min_samples_split*: 5  *GHOST criteria:* 0.25 |
| RF balanced  (Python 3.9 - sklearn lib) | *max_depth:* [5, 8, 15, 25, 30]  *min_samples_leaf*: [1, 2, 5, 10]  *GHOST criteria:* [0:1,0.05] | *max_depth:* 15  *min_samples_split*: 5  *GHOST criteria:* 0.50 | *max_depth:* 15  *min_samples_split*: 5  *GHOST criteria:* 0.50 |
| LDA  (R 4.1 – MASS lib) | - | - | - |


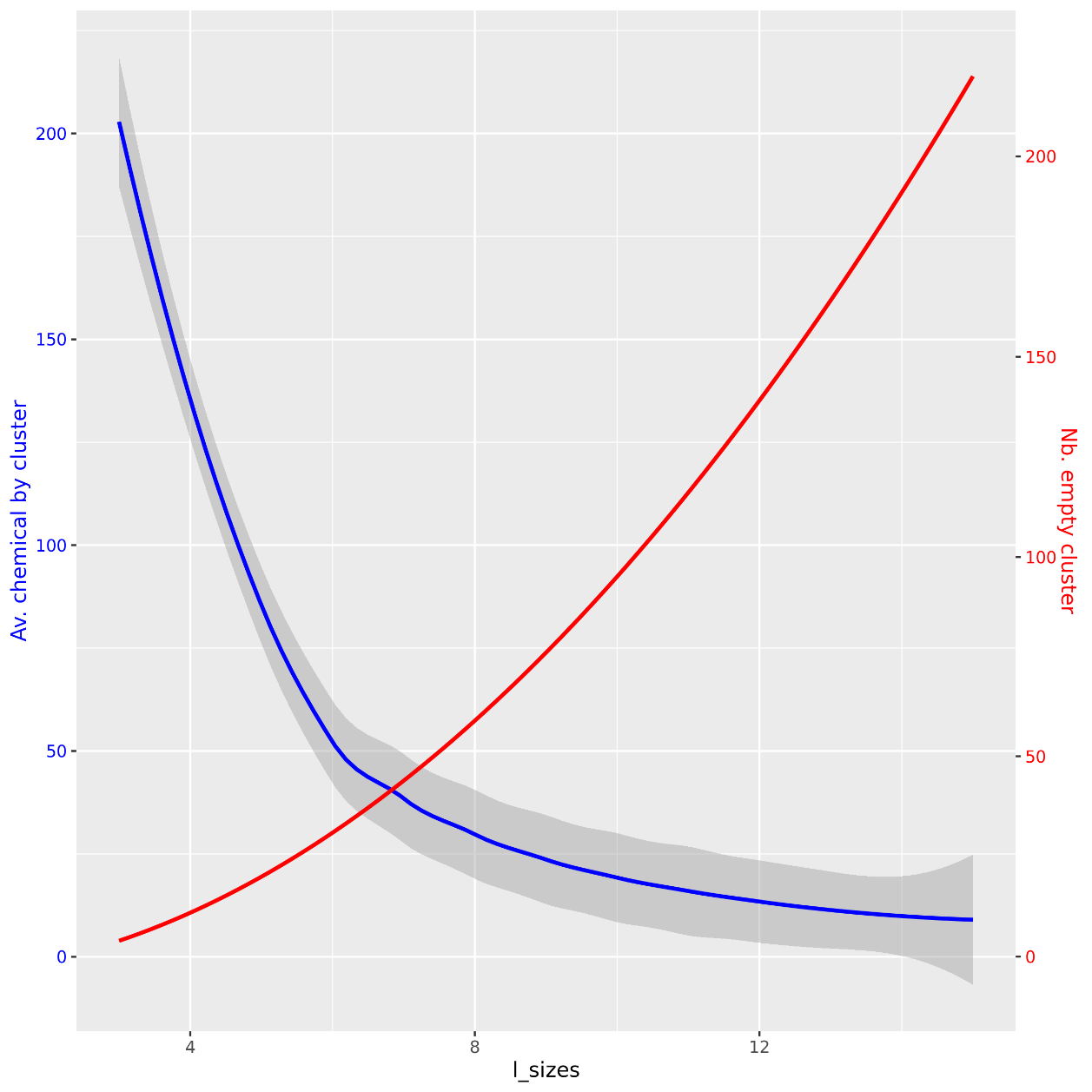


**Figure S1:** Self-Organizing Map optimization. Projection of the number of empty cluster and the average number of chemicals by cluster by size of the SOM.


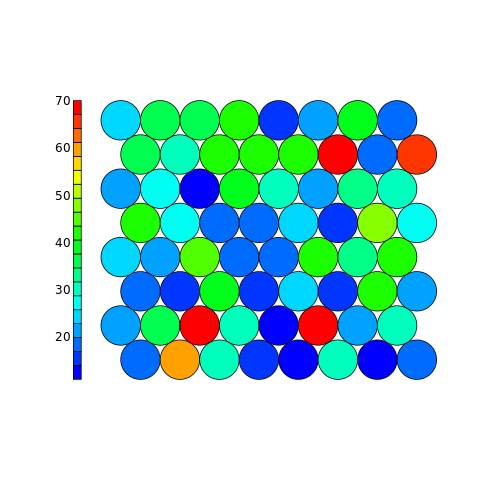


**Figure 2:** Structure-based SOM on the 1925 structure-curated chemicals from chemicals tested with the H295R assay, including 64 clusters in total, colored based on the number of chemicals included in each cluster.
